# Flemish population-based cancer screening programs: impact of COVID-19 related shutdown on short-term key performance indicators

**DOI:** 10.1186/s12885-022-09292-y

**Published:** 2022-02-18

**Authors:** Svetlana Jidkova, Sarah Hoeck, Eliane Kellen, Saskia le Cessie, Mathijs C. Goossens

**Affiliations:** 1 Department of Public Health and Primary Care, Ghent University, Ghent, Belgium; 2 Centre for Cancer Detection, Ruddershove, Bruges, Belgium; 3 Family Medicine and Population Health (FAMPOP), Faculty of Medicine and Health Sciences, University of Antwerp, Antwerp, Belgium; 4 University Hospital Leuven, Campus St. Rafael, Kapucijnenvoer, Leuven, Belgium; 5 Department of Clinical Epidemiology, Department of Biomedical Data Sciences, Leiden University Medical Centre, Leiden, Netherlands; 6 Vrije Universiteit Brussel, Brussels, Belgium

**Keywords:** Cancer screening, Cervical, Breast, Colorectal, COVID-19, Interruption of screening, Participation

## Abstract

**Background:**

Many breast, colorectal, and cervical cancer screening programs were disrupted due to the COVID-19 pandemic. This study aimed to estimate the short-term impact of the temporary shutdown (from March until May- June) of the cancer screening programs invitations in Flanders (Belgium) by looking at invitation coverage, percentage of people screened after invitation and the screening interval.

**Methods:**

Yearly invitation coverage was calculated as the number of people who received an invitation, as a proportion of the people who should have received an invitation that year. Weekly response to the invitation was calculated as the number of people who were screened within 40 days of their date of invitation, as a percentage of the people who received an invitation that week (as a proxy for willingness to screen). Weekly screening interval was calculated as the mean number of months between the current screening and the previous screening of all the people who screened that week. The two last indicators were calculated for each week in 2019 and 2020, after which the difference between that week’s value in 2020 and 2019 with 95% confidence intervals. Results of these two indicators were also analysed after stratification for gender, age and participation history.

**Results:**

Invitation coverage was not impacted in the colorectal and cervical cancer screening program. In the breast cancer screening program invitation coverage went down from 97.5% (2019) to 88.7% (2020), and the backlog of invitations was largely resolved in the first six months of 2021. The willingness to screen was minimally influenced by COVID-19. The breast cancer screening program had a temporary increase in screening interval in the first months following the restart after COVID-19 related shutdown, when it was on average 2.1 months longer than in 2019.

**Conclusions:**

Willingness to screen was minimally influenced by COVID-19, but there may be an influence on screening *coverage* because of lower invitation coverage, mainly for the for breast Cancer Screening Program. The increases in screening intervals for the three Cancer Screening Program seem reasonable and would probably not significantly increase the risk of delayed screening cancer diagnoses. When restarting a Cancer Screening Program after a COVID-19 related shutdown, monitoring is crucial.

**Supplementary Information:**

The online version contains supplementary material available at 10.1186/s12885-022-09292-y.

## Background

In January 2020, the World Health Organization (WHO) confirmed that a novel coronavirus, SARS-CoV2, was the cause of a cluster of pneumonia cases in Wuhan City, China,. This disease would later become known as COVID-19. [1] First cases were reported in Belgium at the beginning of February 2020.

On the 17^th^ of March 2020, the different levels of Belgian government implemented strict national and regional measures to stop the spread of COVID-19. Strict social distancing measures were imposed, non-essential international travel was prohibited, non-essential shops and schools were closed and home-office was made the standard. People were only allowed to leave their home for exercise, essential shopping (e.g. supermarket or pharmacy) or to receive healthcare. All elective medical procedures and treatments were temporarily suspended, including the cancer screening programs. In May 2020, restrictions eased and non-urgent health services, such as elective surgeries and the cancer screening programs, resumed. Belgium was hit hard by COVID-19 in 2020 with 649,505 confirmed cases and 19,720 deaths, representing a crude cumulative death rate of 1704 per million inhabitants. (reference: https://www.sciensano.be/nl/gezondheidsonderwerpen/coronavirus). The epidemic presented two waves; the first wave was observed from the 10th of March to the 21st of June. After an interwave period, the second wave started on the 31st of August, and was still ongoing by end of 2020. Belgium attracted attention internationally due to a high COVID-19 related mortality during the first wave. (reference: https://www.healthybelgium.be/en/health-status/covid-19-crisis/covid-19-mortality).

COVID-19 has a direct effect on morbidity and mortality, but can also indirectly increase disease burden by complicating care for other diseases. In the case of breast, cervical and colorectal cancer, the temporary shut-down of treatment facilities and the delayed diagnosis, caused by disruption of cancer screening programs invitations may have increased disease burden.

The goal of cancer screening is to detect and treat cancer in an early stage, leading to a reduction in cancer–related mortality. A temporary shutdown of a screening program should remain as brief as possible. For colorectal cancer screening delay beyond 4–6 months would significantly increase advanced colorectal cancer cases [2]. Recent research showed that a two-month prolongation of the interval in a mammographic screening program already produced a significant increase in node-positive breast cancers. SA restart of a screening program obviously needs to meet a minimum requirement for infection prevention. [3]

The aim of this report was to analyse the short-term impact of COVID-19 and the shutdown of invitations of the three cancer screening programs throughout 2020. To measure impact, we looked at the effect on three short-term Key Program Indicators of a cancer screening program: invitation coverage, percentage of people that were screened within 40 days after their invitation, and the screening interval.

## Methods

In Flanders (the Northern region of Belgium), three population-based cancer screening programs (CSP) exist:

- The breast CSP, offers a double read mammogram every two years among women aged 50–69 years old; the invitation letter contains a pre-fixed appointment.
- The colorectal CSP, offers an FIT (Fecal Immunochemical Test) every two years among men and women aged 50–74 years old.
- The cervical CSP, promotes to have a Papanicolaou (PAP) smear taken, every three years among women aged 25–64 years old. The program only invites women who did not had a PAP smear taken on her spontaneous initiative, or that of her physician (opportunistically screening).

The screening programs were described extensively previously [4, 5]. In short, the screening programs use a centralized invitation procedure: all invitation letters and information leaflets are sent out by the Centre for Cancer Detection, by post. Participating in the breast and colorectal CSP is free of charge. The target population is based on current age, vital status and place of residence, and is updated throughout the year, as the demographic situation daily evolves. According to the invitation strategy of the screening program, individuals in the target population should receive a new invitation 24 month after their last screening (or after their last invitation in case of no screening) for breast CSP and colorectal CSP, and after 36 months after their last invitation for the cervical CSP.The costs of participating in cervical CSP is regulated by the Belgian health care system which means a certain percentage of the total cost are out-of-pocket. This also applies to the cost of diagnostic assessment following a positive screening.

Data from the three screening programs are recorded in an online screening database. It contains data on individual level for the entire target population of the CSP’s, is heavily encrypted and holds information such as date of birth, gender and invitation and participation history. This allows all Key Program Indicators (invitation coverage, percentage screened within 40 days and screening interval) to be calculated on an individual level.

In order to use available time-slots efficiently, future screening behaviour is predicted based on pparticipation history (for the breast CSP and colorectal CSP). There are four categories for participation history:

- Type 1, participated in the last round.
- Type 2, first-time invitees.
- Type 3, participated before, but not in the last round.
- Type 4, never participated.

All methods were carried out in accordance with relevant guidelines and regulations.

### Shutdown periods of the programs

We used week number according to the **ISO-8601**standard, with weeks starting on Monday.

Breast CSP: From 23 March 2020 (week 13) to 28 June 2020 (week 26).

Colorectal CSP: From 22 March 2020 (week 12) to 23 May 2020 (week 20)-and from 15 November 2020 (week 46) to 28 November 2020 (week 47).*The (colorectal) program was also briefly suspended in November 2020 (second COVID-19 wave in Belgium). This was done at the request of the anesthesiologists because their skills are required during diagnostic assessment of a positive screening (full colonoscopy including sedation) but they were experiencing extremely high workload due to the second wave.”*

Cervical CSP: From 22 March 2020 (week 12) to 23 May 2020 (week 20).

### Invitation coverage

The part of the target population that should receive an invitation during a particular year is called “population to be invited year xxxx”; it is determined on the first of January of each year. The invitation coverage was calculated, for 2018, 2019 and 2020, as follows: the number of people who received an invitation, as a proportion of the *population to be invited year xxxx*. Hence, the invitation coverage will never attain 100%, since valid reasons for not sending an invitation are death or moving out of Flanders after the first of January.

### Percentage of people screened within 40 days after invitation

The percentage of people that were screened within 40 days after their invitation was used a proxy of willingness to screen. Since in 2019, the majority of participants were screened within 40 days after invitation (75% for colorectal and 95% for breast CSP), 40 days was used as benchmark.

Firstly, the percentage of people screened 40 days after invitation was calculated for each week of 2019 and 2020 as follows: the number of people who received an invitation that week and were screened within 40 days, as a percentage of the people who received an invitation that week.

Secondly, the difference in between 2020 and 2019 was calculated per week, with 95% confidence intervals (CI) which were based on the asymptotic normal distribution. This indicator is not calculated for cervical CSP, as the program only invites non-responders.

Analyses were repeated after stratification for gender (in the case of colorectal CSP), age (5 years age groups) and participation history (Type 1-Type 4). Stratification for age is potentially more important for the colorectal CSP: the eligible population of the Flemish colorectal CSP gradually extended from the 56 (2013) to 50 years (2020). In 2019, the starting age was 51 and in 2020 50 years. The younger age group consists of 95% first-invitees (first rounds) in 2020, compared to 65% in 2019.

### Screening interval

The screening interval was calculated for each person screened in 2019 and 2020 as the number of months between the current screening date, and his or her previous screening date. Mean screening intervals per week, with 95% CI were calculated. Regarding cervical screening, both PAP smears taken after opportunistic screening, and the ones taken after an invitation for the screening program were taken into account. The difference between 2020 and 2019 mean screening interval per week was calculated, with corresponding 95% CI. Analyses were repeated after stratification for gender (in the case of colorectal CSP), age (5 years age groups) and participation history (Type 1-Type 4 for breast and colorectal CSP).

## Results

### Invitation coverage

Table 1 shows the invitation coverage per CSP in 2018–2020.

**Table 1:** Invitation coverage for cancer screening programs

Year	To be invited	Invited	Not invited	
	N	N (%)	N (%)	
**Breast CSP**				
2018	393,890	386,829 **(98.2)**	7,061 (1.8)	
2019	391,764	381,819 **(97.5)**	9,945 (2.5)	
2020	391,838	347,404 **(88.7)**	44,434 (11.3)	
**Colorectal CSP**				
2018	663,763	647,954 **(97.6)**		15,809 (2.4)
2019	848,266	828,266 **(97.7)**		19,729 (2.3)
2020	883,376	869,814 **(98.5)**		13,462 (1.5)
**Cervical CSP**				
2018	238,932	228,694 (**95.7%)**		10,238 (4.3%)
2019	294,006	280,044 **(95.3%)**		13,962 (4.7%)
2020	301,652	320,767 **(94.1%)**		15,653 (5.9%)

In the breast CSP, the invitation coverage was markedly lower in 2020 than in previous years (88.1% in 2020 versus 97.5% in 2019). The backlog consisted of 44,434 women, of whom 61.8% (*n* = 27,441) were invited in the first three months of 2021, 20.3% (*n* = 9,031) in the second three months (April – June) of 2021 and 0.4% (*n* = 194) participated without an invitation (self-registration). The remaining 17.5% (*N* = 7,768) have not yet been invited. These 7,768 women represent 2.0% of the total group of women to be invited in 2020.

For the colorectal CSP, the invitation coverage remained stable in 2020 (98.5% in 2020 vs 97.7 in 2019). The invitations that were not sent in March–May 2020 were all sent in June-July 2020. The invitations that were not sent at the beginning of November 2020 were all sent in November—December 2020.

The invitation coverage for the cervical CSP also remained stable (94.1% in 2020 vs 95.3% in 2019). All backlog invitations of the shutdown period were sent in June 2020.

### Percentage of people screened within 40 days after invitation

**Fig. 1:**
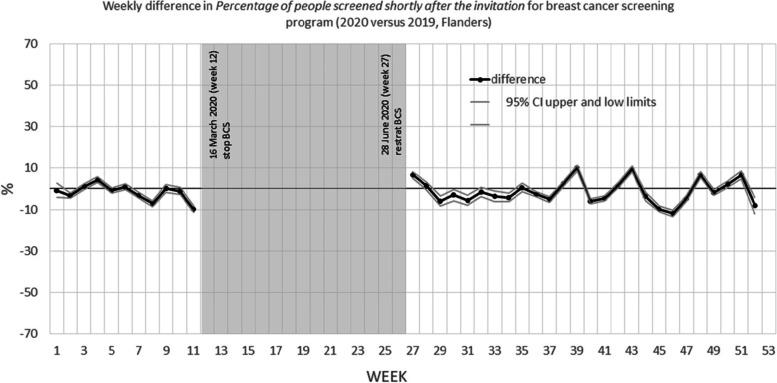
Weekly difference in percentage of people screened within 40 days after the invitation for breast cancer screening program (2020 versus 2019, Flanders)

**Fig. 2:**
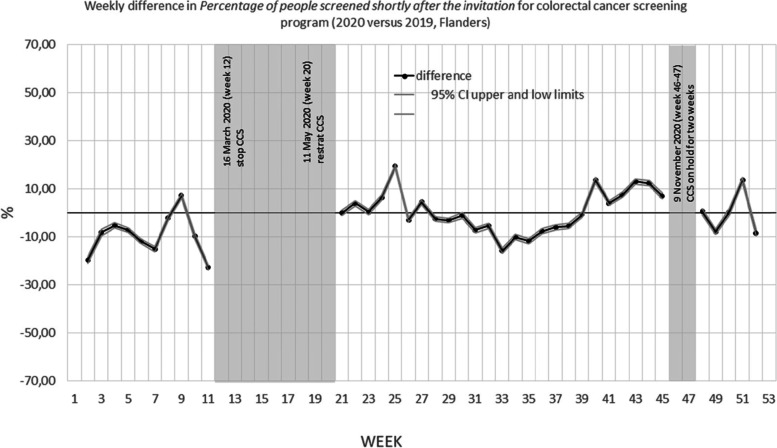
Weekly difference in Percentage of people screened within 40 days after the invitation for colorectal cancer screening program (2020 versus 2019, Flanders)

Figures 1 and 2 show the weekly difference in percentage of people that were screened within 40 days after their invitation between 2020 and 2019. The grey areas are the periods during which no mammograms were performed (breast CSP) or no invitations were sent (colorectal CSP) due to COVID-19 shutdown of the programs.

Table 2 shows that for the breast CSP, the percentage of individuals screened within 40 days in 2020, was 1.0 percent lower after the restart of the programme (from July, 5 (week 27) until the end of the year) than in 2019. When the figures were made separately per age group and participation history (see figures in annex), similar patterns were observed, except for the Type 1 participation history (participated last round). These women participated less often: after the restart of the program their percentage screened within 40 days was 4.0% lower than in 2019.

**Table 2:** Difference in percentage of people screened within 40 days after the invitation for breast cancer screening program (2020 versus 2019, Flanders)

	January, 1 (week 1)– December,31 (week 52)	January, 1 (week1)-March 21 (week11)	July, 5 (week 27) – December, 31 (week 52)	July, 5 (week 27) -November, 28 (week 47)
	(entire year)	(before first COVID-19 shutdown)	(after the restart of the program)	(first 20 weeks after restart of the program)
	% (95% CI)	% (95% CI)	% (95% CI)	% (95% CI)
All invitation	-1.0 (-1.3; -0.8)	-1.4 (-1.9; -1.0)	-1.0 (-1.3; -0.7)	-1.6 (-2.0; -1.3)
Stratified by age				
50–54	-0.5 (-1.0; -0.1)	-2.9 (-3.7; -2.1)	0.4 (-0.1; 1.0)	0.0 (-0.6; 0.6)
55–59	-1.7 (-2.2; -1.2)	-4.0 (-4.9; -3.1)	-0.8 (-1.4; -0.2)	-1.7 (-2.4; -1.0)
60–64	-1.0 (-1.5; -0.6)	-0.3 (-1.1; 0.6)	-1.9 (-2.5; -1.3)	-3.2 (-3.8; -2.5)
65–69	-1.5 (-2.0; -1.0)	-0.5(-1.4; 0.4)	-2.1 (-2.8; -1.4)	-2.3 (-3.0; -1.6)
Stratified by screening history				
Type 1	-3.0 (-3.3; -2.7)	0.1 (-0.4; 0.6)	-4.0 (-4.4; -3.7)	-4.4(-4.8; -4.0)
Type 2	-1.1 (-1.8; -0.4)	-2.8 (-3.9; -1.6)	0.6 (-0.3; 1.5)	-0.3 (-1.2; 0.7)
Type 3	-1.8 (-2.4; -1.2)	-1.1 (-2.2; -0.1)	-2.2 (-3.0; -1.4)	-2.5(-3.3; -1.6)
Type 4	0.1 (-0.2; 0.3)	-0.9 (-1.4; -0.4)	0.7 (0.3; 1.0)	0.3 (-0.1; 0.7)

For the colorectal CSP, the percentage of individuals screened within 40 days in 2020 was 10.2% lower than in 2019 in the period before the first invitation shutdown (from the beginning of the year until March, 21 (week 11)) (Table 3). After the first re-start of the program (from May, 24 (week 21) until November, 14 (week 45), the overall percentage of individuals screened within 40 days was 1.0% higher. Interestingly, this overall increase is the result of a small decrease in every age category, while the 70–74 year old’s have a 20% higher uptake rate. After the second re-start of the program (from November, 29 (week 48) until the end of the year) the overall difference in uptake was 0.3% higher, but in this period the oldest age group has a -16,20% lower uptake rate. Stratified by participation history, the differences in participation seem to be the largest for type 1 (also participated in last round). See Table 3.

**Table 3:** Difference in percentage of people screened within 40 days after the invitation for colorectal cancer screening program (2020 versus 2019, Flanders)

	January, 1 (week 1)– December,31 (week 52)	January, 1 (week1)-March 21 (week 11)	May, 24 (week 21) November, 14 (week45)	November, 29 (week 48) – December, 31 (week52)
	(entire year)	(before first COVID-19 shutdown)	(after first restart of the program)	(after second restart of the program)
	% (95% CI)	% (95% CI)	% (95% CI)	% (95% CI)
All invitation	-2.6 (-2.8; 2.5)	-10.2 (-10.5; -9.9)	1.0 (0.8; 1.2)	0.3 (-0.2; 0.8)
Stratified by gender				
Men	-3.0 (-3.3; -2.8)	-10.9 (11.3; -10.5)	1.0 (0.7; 1.3)	-0.2 (-0.9; 0.5)
Women	-2.2 (-2.4; -2.0)	-9.6 (-10.0; -9.1)	1.0 (0.8; 1.4)	0.8 (0.1; 1.5)
Stratified by age				
50–54	-3.9 (-4.2; -3.6)	-7.8 (-8.3; -7.3)	-1.4 (-1.8; -1.1)	-4.4 (-6.5; -2.4)
55–59	-1.1 (-1.4; -0.7)	-8.5 (-9.3; -7.6)	-2.7 (-3.1; -2.4)	15.0 (14.2; 15.9)
60–64	-0.8 (-1.2; -0.5)	0.9 (0.2; 1.5)	-1.3 (-1.8; -0.9)	-7.1 (-8.1; -6.0)
65–69	-2.4 (-2.9; -2.0)	-6.0 (-6.9; -5.2)	-1.1 (-1.7; -0.5)	1.9 (0.6; 3.2)
70–74	-0.9 (-1.3; -0.5)	-24.0 (-25.0; -23.3)	19.9 (19.4; 20.5)	-16.2 (-17.4; -15.0)
Stratified by screening history				
Type 1	-3.9 (-4.1; -3.6)	-11.5 (-12; -11.0)	1.5 (1.2; 1.8)	-6.6 (-7.3; -5.8)
Type 2	-3.7 (-4.0; -3.4)	-7.2 (-7.7; -6.8)	-1.5 (-1.8; -1.2)	-6.2 (-7.8; -4.7)
Type 3	-3.1 (-4.2; -2.1)	-10.9 (-12.8; -9.1)	1.6 (0.2; 3.1)	2.3 (-0.3; 4.8)
Type 4	-0.1 (-0.3; 0.0)	-2.3 (-2.6; -2.0)	0.7 (0.5; 0.9)	0.6 (0.2; 1.0)

### Screening interval

**Fig. 3:**
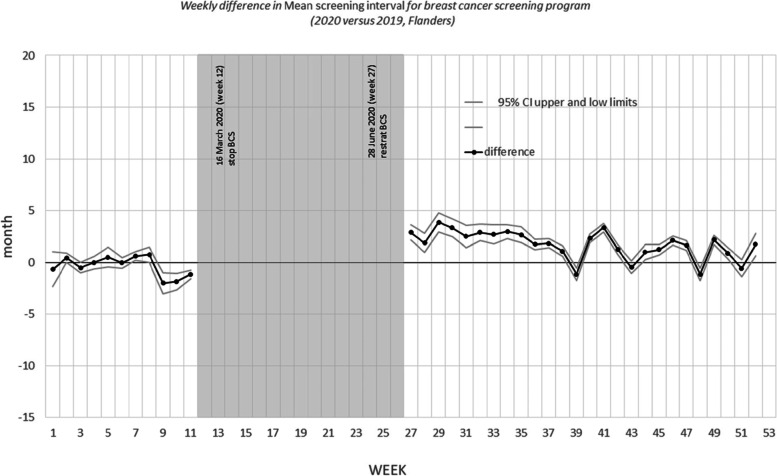
Weekly difference in mean screening interval for breast cancer screening program (2020 versus 2019, Flanders)

**Fig. 4:**
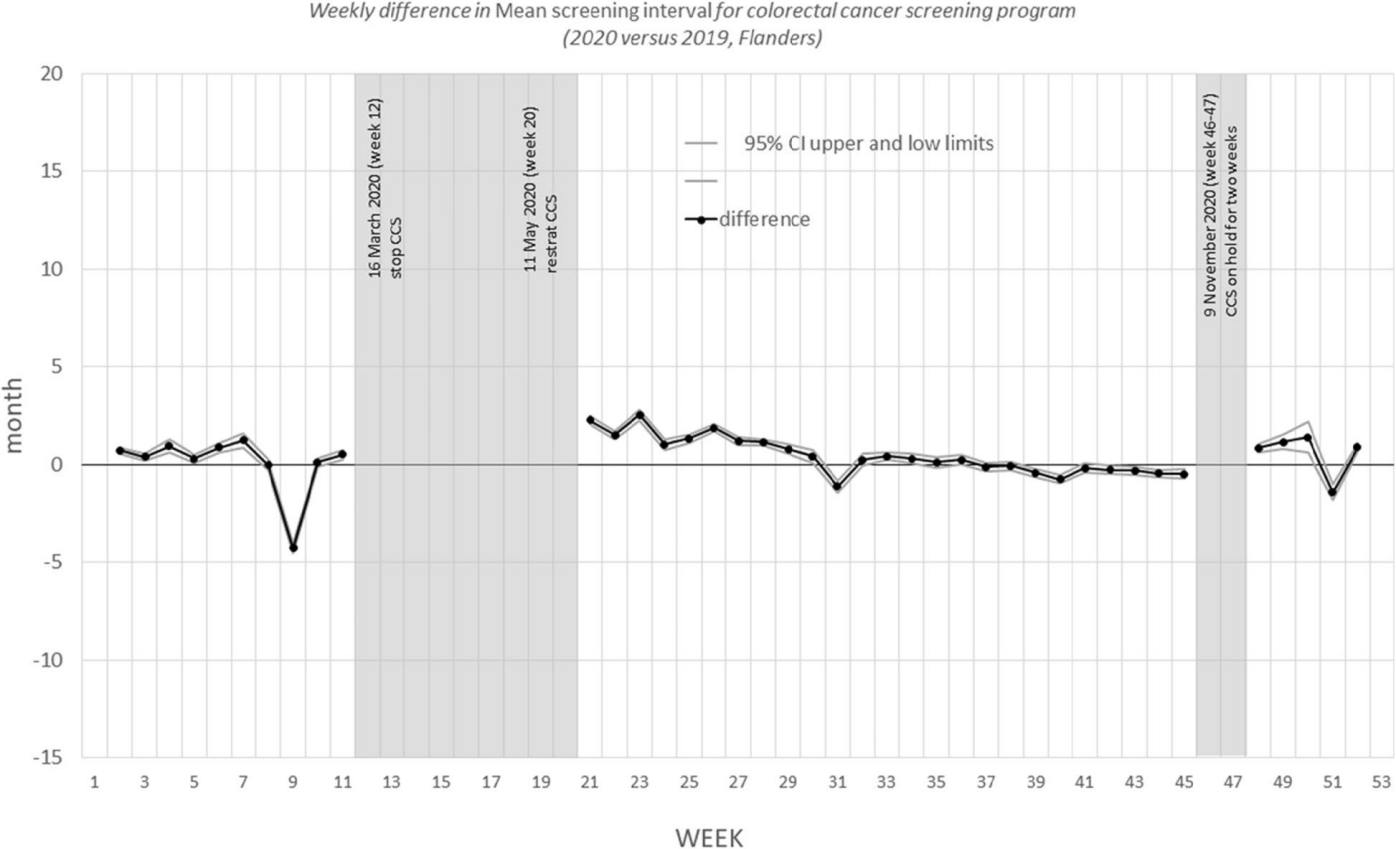
Weekly difference in mean screening interval for colorectal cancer screening program (2020 versus 2019, Flanders)

**Fig. 5:**
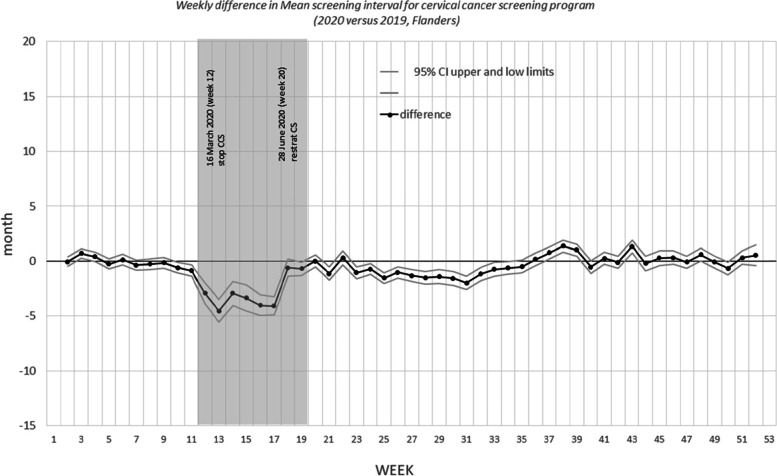
Weekly difference in mean screening interval for cervical cancer screening program (2020 versus 2019, Flanders)

Figures 3, 4, and 5 show the differences in the mean screening interval per week. The grey areas are the periods during which no mammograms were performed (breast CSP) or no invitations were sent (colorectal CSP and cervical CSP) due to COVID-19 shutdown of the programs.

For the breast CSP (Fig. 3), the screening interval increased temporarily after the restart of the program: in the period of July, 5 (week 27) to November, 28 (week 47) of 2020 it was on average 2.1 months longer than in 2019. After November, 28 the screening interval return back to normal. When this analysis was done separately after stratifying for age group and participation history, the observed patterns were similar (see figures in annex).

For the colorectal CSP (Fig. 4), the screening interval increased in the first weeks after the first restart of the program (from May, 24 (week 21) – until July,25 (week 29)), normalizes and increased again in the first weeks after the second restart (November, 29 (week 48) until – December, 19 (week 50)). The largest screening intervals measured in 2020 were in the week of June, 7 (week 23) (28,55 months) and that of December, 19 (week 50) (30,29 months). For entire 2020 the difference in mean screening interval between 2020 and 2019 was 0,50 months () (Table 4). The results did not change after stratification by gender. The screening interval differs by age: all age categories report a higher screening interval difference in the period from May, 24 (week 21) – until July,25 (week 29)),, but only the 65–69 and 70–74 years old also in the period from October, 11 (week 41) until November, 14 (week -45) (before the second invitation shutdown). After the second shutdown period (from November, 29 (week 48) until the end of the year): the mean difference in screening interval increases with age: 0.1 months for the 55–59 years old, 2.7 months for the 60–64, 2.5 months for the 65–69 and 3.6 months for the 70–74 years old. Stratified by participation history, the mean difference in screening interval seems to be the largest for type 3 (participated before but not in last round). (See Table 5).

**Table 4:** Difference in mean screening interval for breast cancer screening program (2020 versus 2019, Flanders)

	January, 1 (week 1)– December,31 (week 52)	January, 1 (week1)-March 21 (week 11)	July, 5 (week 27) – December, 31 (week 52)	July, 5 (week 27) -November, 28 (week 47)
	(entire year)	(before first COVID-19 shutdown)	(after the restart of the program)	(first 20 weeks after restart of the program)
	months (95% CI)	months (95% CI)	months (95% CI)	months (95% CI)
All screened	1.3 (1.4; 1.2)	-0.2 (-0.0; -0.3)	1.9 (2.0; 1.8)	2.1 (2.2; 2.0)
Stratified by age				
50–54	1.3 (1.2; 1.4)	0.1 (0.0; 0.3)	1.5 (1.4; 1.6)	1.6 (1.5; 1.8)
55–59	1.4 (1.3; 1.6)	0.1 (-0.2; 0.4)	1.9 (1.7; 2.1)	2.2 (2.0; 2.4)
60–64	1.2 (1.1; 1.4)	-0.3 (-0.7; 0.1)	2.0 (1.7; 2.2)	2.2 (1.9; 2.4)
65–69	1.0 (0.8; 1.3)	-0.9 (-1.4; -0.5)	2.1 (1.8; 2.4)	2.3 (1.9; 2.6)
Stratified by screening history^a^				
Type 1	1.4 (1.4; 1.4)	0.0 (0.0; 0.0)	1.9 (1.9; 1.9)	2.1 (2.0; 2.1)
Type 3	2.0 (0.8; 1.3)	-1.8 (-3.1; -0.6)	1.3 (0.5; 2.2)	4.3 (3.4; 5.2)

^a^cannot be calculated for type 2 (first invitation) or type 4 (never participated in the past). In the period 12–27 weeks no invitations were send out due to lock down

**Table 5:** Difference in mean screening interval for colorectal cancer screening program (2020 versus 2019, Flanders)

	January, 1 (week 1)– December,31 (week 52)	January, 1 (week1)-March 21 (week 11)	May, 24 (week 21) November, 14 (week 45)	November, 29 (week 48) – December, 31 (week52)
	(entire year)	(before first COVID-19 shutdown)	(after first restart of the program)	(after second restart of the program)
	months (95% CI)	months (95% CI)	months (95% CI)	months (95% CI)
All screened	0.5 (0.5; 0.5)	0.0 (0.0; 0.1)	0.7 (0.6; 0.7)	0.5 (0.4; 0.6)
Stratified by gender				
men	0.6 (0.5; 0.6)	0.1 (0.0; 0.2)	0.7 (0.6; 0.8)	0.5 (0.3; 0.7)
women	0.5 (0.4; 0.5)	0.0 (-0.1; 0.1)	0.6 (0.6; 0.7)	0.5 (0.3; 0.7)
Stratified by age^a^				
55–59	0.3 (0.3; 0.3)	-0.2 (-0.2; -0.1)	0.4 (0.3; 0.4)	0.1 (0.0; 0.2)
60–64	0.6 (0.5; 0.7)	-0.7 (-0.9; -0.6)	1.0 (0.9; 1.1)	2.7 (2.3; 3.1)
65–69	0.9 (0.8; 1.0)	0.1 (0.0; 0.3)	1.1 (0.9; 1.2)	2.5 (3.1; 2.0)
70–74	1.1 (1.0; 1.2)	1.2 (1.0; 1.4)	0.1 (0.0; 0.3)	3.6 (3.0; 4.2)
Stratified by screening history^a^				
Type 1	0.6 (0.6; 0.6)	0.2 (0.2; 0.3)	0.7 (0.6; 0.7)	0.5 (0.4; 0.6)
Type 3	0.8 (0.6; 0.9)	-0.7 (-0.9; -0.4)	1.3 (1.1; 1.5)	2.2 (1.8; 2.6)

^a^cannot be calculated for age 50–54 (first invitation), type 2 (first invitation) or type 4 (never participated in the past). In the period 12–20 weeks and 46–47 weeks no invitations were send out due to lock down

For the cervical CSP, the mean screening interval between the last and previous PAP smears taken in 2019 and 2020, oscillated around null, except for the period from March, 22 (week 12) until May, 23 (week 20). During the shutdown period of the CSP, the screening period was lower than in 2019, as the opportunistic screening and follow up smears continued to be taken. During that period, only half the number of PAP smears was taken, compared to the same period in 2019 (*n* = 20,217 vs 45,667). Starting from May, 24 (week 21), the number of PAP smears increased rapidly and the mean screening interval oscillated again around null from July, 26 (week 30) onwards. Similar results were seen for all age categories, when the analysis was stratified for age (Table 6).

**Table 6:** Difference in mean screening interval for cervical cancer screening program (2020 versus 2019, Flanders)

	January, 1 (week 1)– December,31 (week 52)	January, 1 (week1)-March 21 (week 11)	March, 22 (week 12)- May, 23 (week 20)	May, 24 (week 21) December, 31 (week52)
	(entire year)	(before COVID-19 shutdown)	(during COVID-19 shutdown)	(after restart of the program)
	months (95% CI)	months (95% CI)	months (95% CI)	Months(95% CI)
All women screened	-0.3 (-0.2; -0.4)	-0.1 (-0.2; 0.1)	-2.3 (-2.1; -2.6)	-0.3 (-0.5; -0.2)
Stratified by age				
25–29	0.1 (-0.1; 0.3)	0.3 (-0.1; 0.8)	-1.2( -1.9; -0.0)	0.0 (-0.3; 0.3)
30–34	-0.1 (-0.3; 0.1)	0.4 (0.0; 0.8)	-1.8 (-2.4; -1.1)	-0.3 (-0.6; -0.1)
35–39	0.0 (-0.2; 0.2)	0.5 (0.1; 0.9)	-1.6 (-2.3; -0.9)	-0.2 (-0.5; 0.1)
40–44	-0.2 (-0.4; 0.0)	-0.3 (-0.7; 0.1)	-2.0 ( -2.7; 1.3)	-0.2 (-0.5; 0.1)
45–49	-0.3 (-0.5; -0.1)	-0.6 (-1.0; -0.2)	-2.4 (-3.0; -1.7)	-0.1 (-0.4; 0.2)
50–54	-0.6 (-0.8; -0.4)	-0.5 (-0.9; -0.1)	-3.4 (-4.1; -2.7)	-0.5 (-0.2; -0.7)
55–59	-0.7 (-1.0; -0.5)	-0.4 (-0.8; 0.0)	-3.3 (-4.0; -2.6)	-0.9 (-1.2; -0.6)
60–64	-0.5 (-0.8; -0.2)	0.0 (-0.5; 0.5)	-2.8 (-3.7; -1.9)	-0.7 (-1.0; -0.4)

## Discussion

An effective infection control response to the COVID-19 pandemic is without doubt crucial. There is however also a risk of indirect morbidity from these measures, among others through a disruption of CSP’s. Failing to screen can itself increase cancer–related mortality. When restarting a CSP after a COVID-19 related shutdown, the monitoring of short-term Key Program Indicators such as invitation coverage, willingness to screen and screening interval is therefore crucial. To our knowledge, this is the first study reporting the COVID-19 impact on the Key Program Indicators for three CSP’s, and describing how this impact differs between the different programs.

In **invitation coverage**, most impact was seen in the breast CSP, where the number of appointments was limited allowing for social distancing and additional hygiene measures. At the end of 2020, the backlog was 11.3% (44,434) of the women that should have been invited that year.

All but 17.5% of them (*N* = 7,768) have been invited in the first months of 2021. These 7,768 women represented 2.0% of the total group of women to be invited in 2020.

The colorectal CSP is home based, hence there was no need to scale down the number of invitations. However on demand of the hospital settings, the invitations were put on hold for two periods. The backlog of invitations was not an issue for the colorectal CSP and cervical CSP;the invitations that were put on hold were sent within a couple of weeks after the restart of the programs. The people who did not received an invitation in 2020 (1.5% for colorectal CSP and 4.5% for cervical CSP) were not COVID-19 related, only due to the fact that the denominator includes people who recently died or moved out of Flanders.

Regarding **willingness to screen,** the people who received an invitation after the restart were nearly as likely to screen as before COVID-19 (< 2% mean difference for the breast CSP and < 1% for the colorectal CSP). An exception seems to be the regular screeners (type 1) in the breast CSP. These women were less likely to participate in the weeks following the restart; their biggest drop ( in the period from October, 11 (week 41) until December, 5 (week 48)) comes just after the start of the second wave of infections. [6]

A nation‐wide media campaign ‘Don’t postpone your consultation with your doctor’ was launched to inform the general public that it was safe to consult healthcare providers. Furthermore, the CSP’s added information to the screening letters, positive screening result letters, and on the website to reassure participants that a visit to a physician, hospital or mammographic unit was risk-free due to strict COVID-19 protective measures.

**The screening interval** was increased in the breast CSP by an average of about two months in the first four months after restarting breast cancer screening. This was due to the fact that women who would have been invited during the shutdown would have had a screening interval of exactly 24 months, and they were postponed by a few months. This two month delay could theoretically lead to a small shift in stage distribution. In order to be sure about the effect on stage distribution, we will need to wait until the beginning of 2022, when data from the cancer registry will be made available for these screenings. In the mean time, we can only rely on existing literature on this subject, and unfortunately not all literature points in the same direction. On the one hand a recent study concluded that a one-time delay of up to six months should not impact cancer-specific mortality rates, especially after a swift restart as was the case in Flanders [7] However, other recent research showed that a two-month prolongation of the interval in a breast CSP already produced an increase in node-positive breast cancers[3].

For the colorectal CSP, the screening interval remains acceptable after both invitation-shutdowns, with a mean screening interval of 25.5 months. The screening interval exceeded 26 months in weeks shortly after the invitation-shutdowns. However, results stratified by age indicated that the screening interval increases with age. A recent modelling study indicated for colorectal CSP that, with immediate catch-up screening, the impact of invitation shutdown would be minimized to a relative increase in excess deaths of 0,1% [7, 8] after an invitation shutdown of 6 months (compared to the only 10 weeks and 3 weeks shutdown in the Flemish colorectal CSP). Short screening delays (4–6 months) do not significantly reduce the performance of screening [2]. Corley [9] stated that compared with a follow-up colonoscopy at 8 to 30 days, only a follow-up after 10 months was associated with a higher risk of colorectal cancer and more advanced-stage disease at the time of diagnosis. Patients who are invited to screening (after the shutdown of invitations) were reassured that it was safe to attend and to plan a follow-up appointment when needed. However, it is unknown how many people with a positive FIT test are awaiting an appointment for a follow-up colonoscopy. In February 2021 the Centre for Cancer Detection conducted an online survey among participants with a FIT positive (between 11/2019 until 03/2020) who had not yet undergone a follow-up colonoscopy, to explore the amount of rescheduled or cancelled colonoscopies and to explore the impact of COVID-19 on compliance to colonoscopy.

Minor disruptions to the cervical CSP are unlikely to cause significant increases in cervical cancer burden, given the slow natural history of cervical precancerous lesions and cancer. A model of screening in the United Kingdom showed that a 6-month screening disruption could lead to an increased risk for cervical cancer. However, the authors concluded that the risk of cervical cancer to an average woman who would have attended screening was seven times higher if they had to delay their screening for a whole screening round than if they had to delay screening for only 6 months [10]. In Flanders, 85% of PAP smears are taken by gynaecologists; hence the fact that primary care physicians were overwhelmed by the care for COVID patients had a limited effect on cervical cancer prevention. Furthermore, screening is based on cytology. Globally, the demand for SARS-CoV-2 testing is competing with HPV testing, compounded by a shortage of staff [11]. However, disregarding the risk profile of the individuals not attending might exacerbate pre-existing inequalities in accessing cervical screening. Modelling of cervical screening outcomes before and during the pandemic supports risk-based strategies as the most effective way for screening services to recover. Recovery strategies should verify that those women at highest risk or the most susceptible to cervical cancer are being screened [12]. Unfortunately, our analysis cannot affirm this. Future research should investigate whether or not the eldest women who fell out of the target population missed a screening during the lockdown procedure and/or by the screen-positive due to fear of contracting COVID-19 [13, 14]—might delay cancer screening diagnoses.

For Flanders, post-COVID-19 analyses are required to explore the compliance with follow-up after a positive screening and the staging of the three screening cancers. Reduction in cancer diagnoses has been noted in other European countries, particularly for colon and skin cancers [15, 16]. In Belgium there seems to be a general drop in the number of screening-related samples [17], however only the first months of 2020 are included in this study. The analyses of the impact of the COVID-19 on the number diagnoses and compliance with follow-up after a positive screening for Flanders is ongoing (by the Belgian Cancer Registry).

### Strengths and limitations

Our Key Program Indicators are calculated on patient-level data and measurement bias on data from the screening database is considered to be extremely low thanks to an important number of automated inconsistency checks that are performed when the data are entered. Another major strength of our study is the fact that we are able to present results within one year after the start of the COVID-19 epidemic in Belgium.

The limitations of this study include the absence of data of the follow up examinations for the three CSP’s. Data on the COVID-19 impact on cancer diagnoses and staging are currently still lacking.. A final limitation of our study was the incompleteness of the number of PAP screens after the lockdown period.

## Conclusion

In conclusion, our results show that willingness to screen was minimally influenced by COVID-19, but there may be an influence on screening *coverage* because of lower invitation coverage for breast CSP. The increases in screening interval for the three CSP’s in Flanders seem reasonable and would probably not significantly increase the risk of delayed screening cancer diagnoses. Moreover, a timely referral and treatment after a positive screening result throughout the different waves of the pandemic, is crucial to maintain an effective CSP.

Minor disruptions to the cervical CSP are unlikely to cause a shift in stage, given the slow natural history of cervical precancerous lesions. A stage-shift in the breast and colorectal CSP is unlikely to have been occurred since the increase in screening interval was limited, but cannot be completely discarded. Follow up analysis of the Belgian Cancer Registry are needed to demonstrate whether or not the efforts not compromise diagnostic and cancer services were sufficient.

## Supplementary Information

**Additional file 1.**

## Data Availability

Data on screening participation per program, per year, can be found on the interactive website: https://bevolkingsonderzoek.incijfers.be/. The datasets used and analysed during the current study are available from the corresponding author on reasonable request.

## Abbreviations

CSP: Cancer Screening Program
CI: Confidence Interval
FIT: Fecal Immunochemical Test
PAP: Papanicolaou test
WHO: World Health Organization
