# Supplementary material for: Flemish population-based cancer screening programs: impact of COVID-19 related shutdown on short-term key performance indicators
Source: BMC Cancer. 2022 Feb 18;22:183. doi: 10.1186/s12885-022-09292-y (PMC8853842; doi:10.1186/s12885-022-09292-y)
Supplement: Supplementary file 1 — Additional file 1. [file 12885_2022_9292_MOESM1_ESM.docx]

| Figure 1 Weekly difference in percentage of people screened within 40 days after the invitation for **breast** cancer screening program, by age (2020 versus 2019, Flanders). | |
| --- | --- |
| 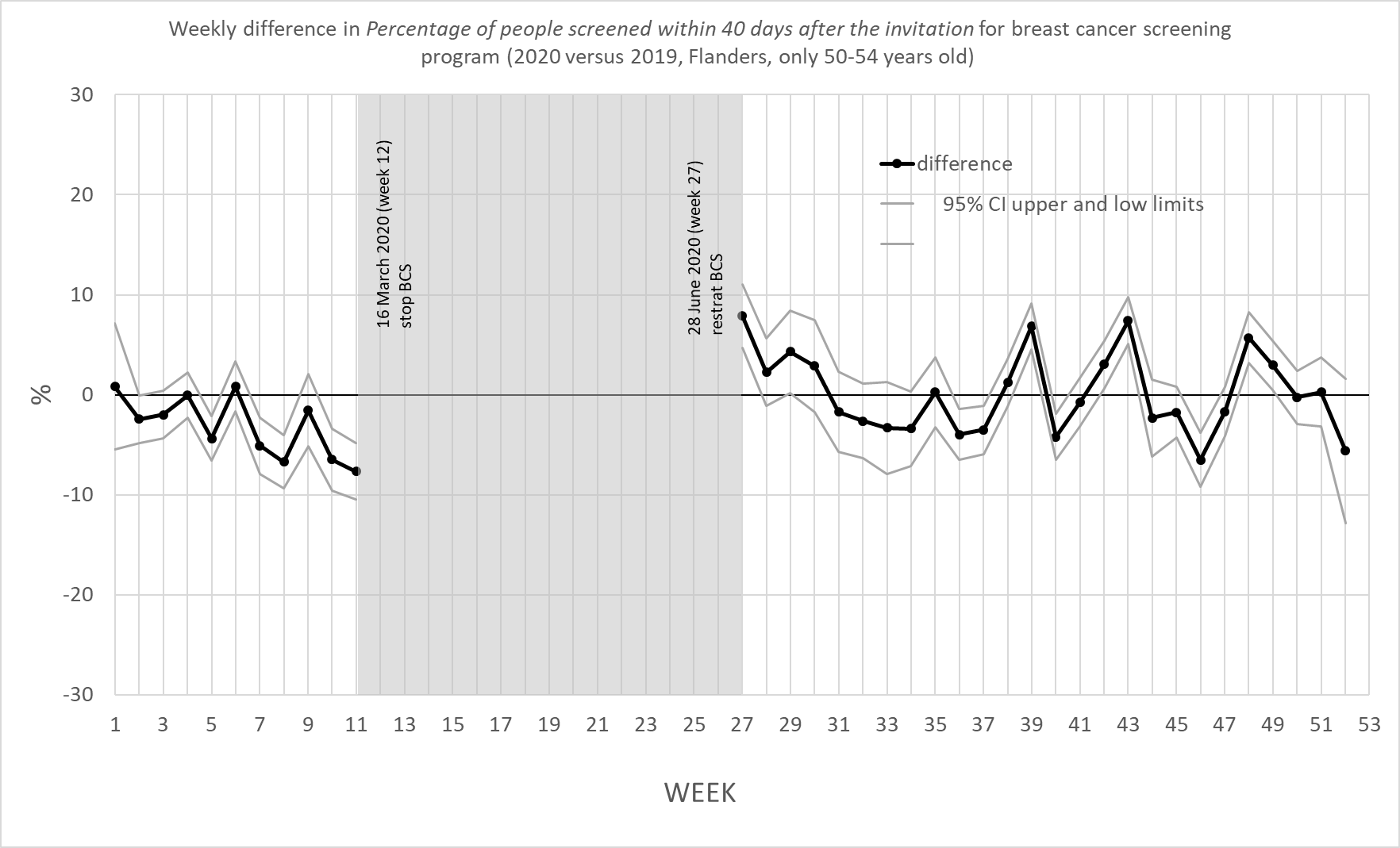 | 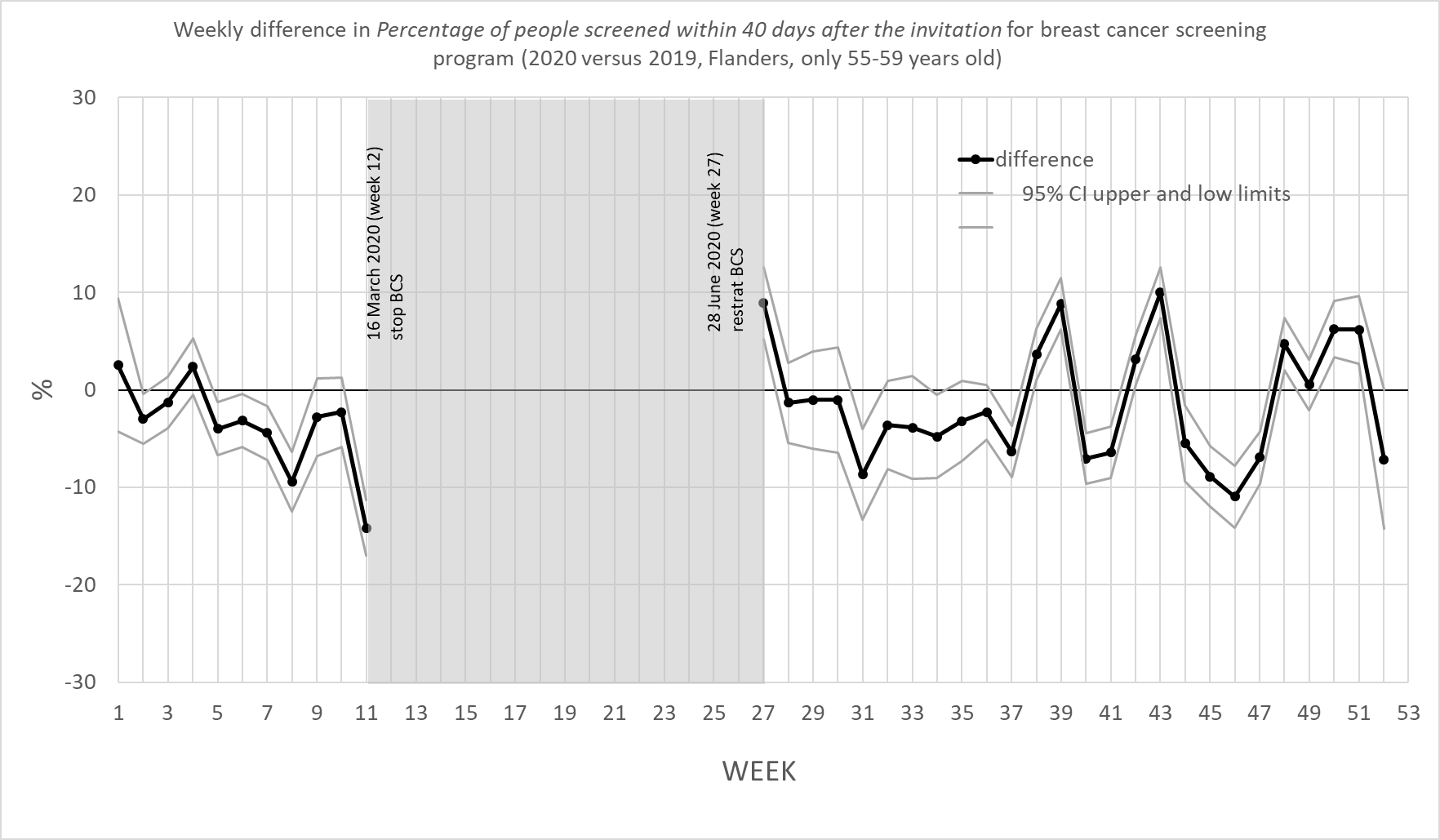 |
| a. 50-54 years old | b. 55-59 years old |
| 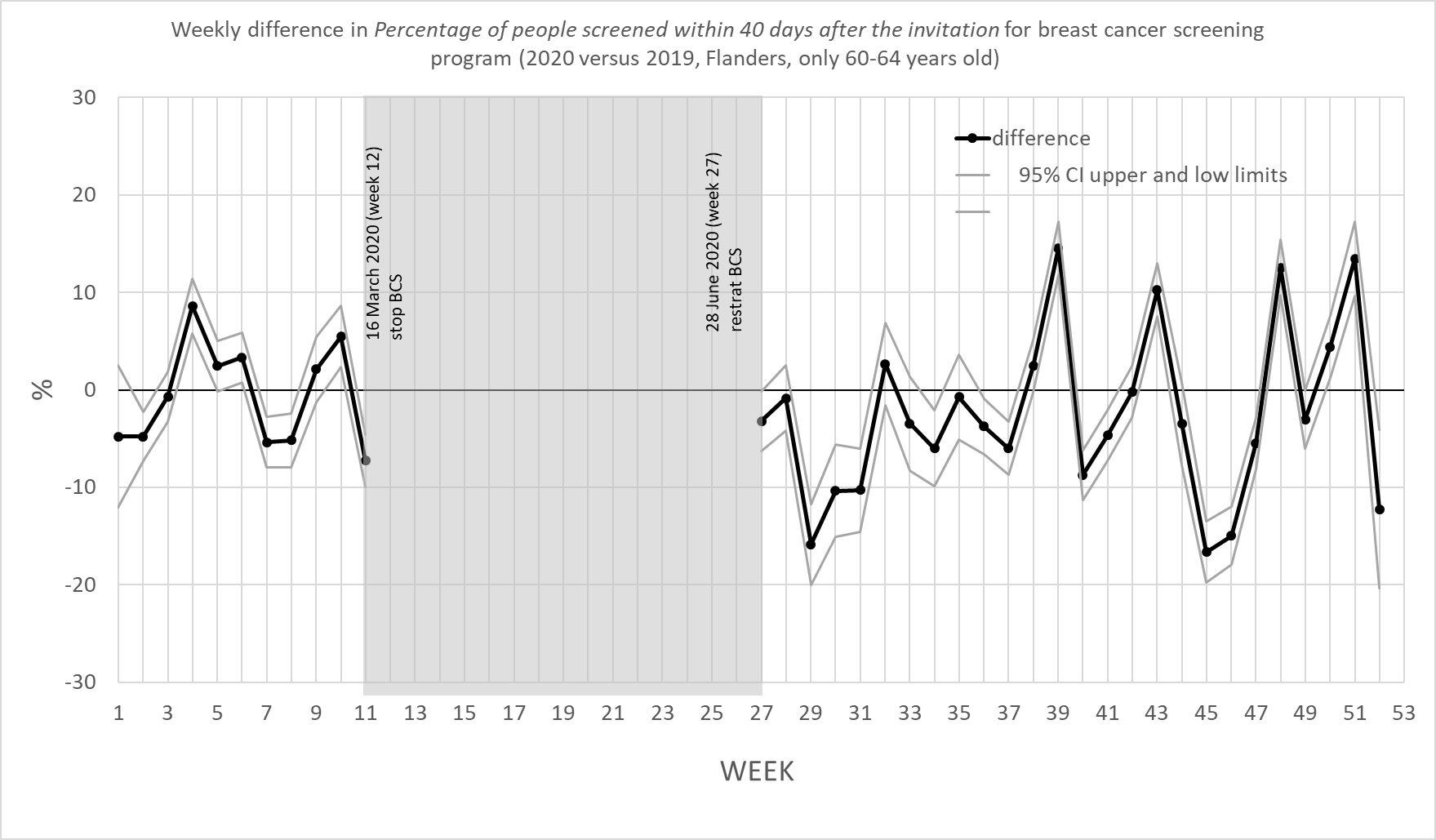 | 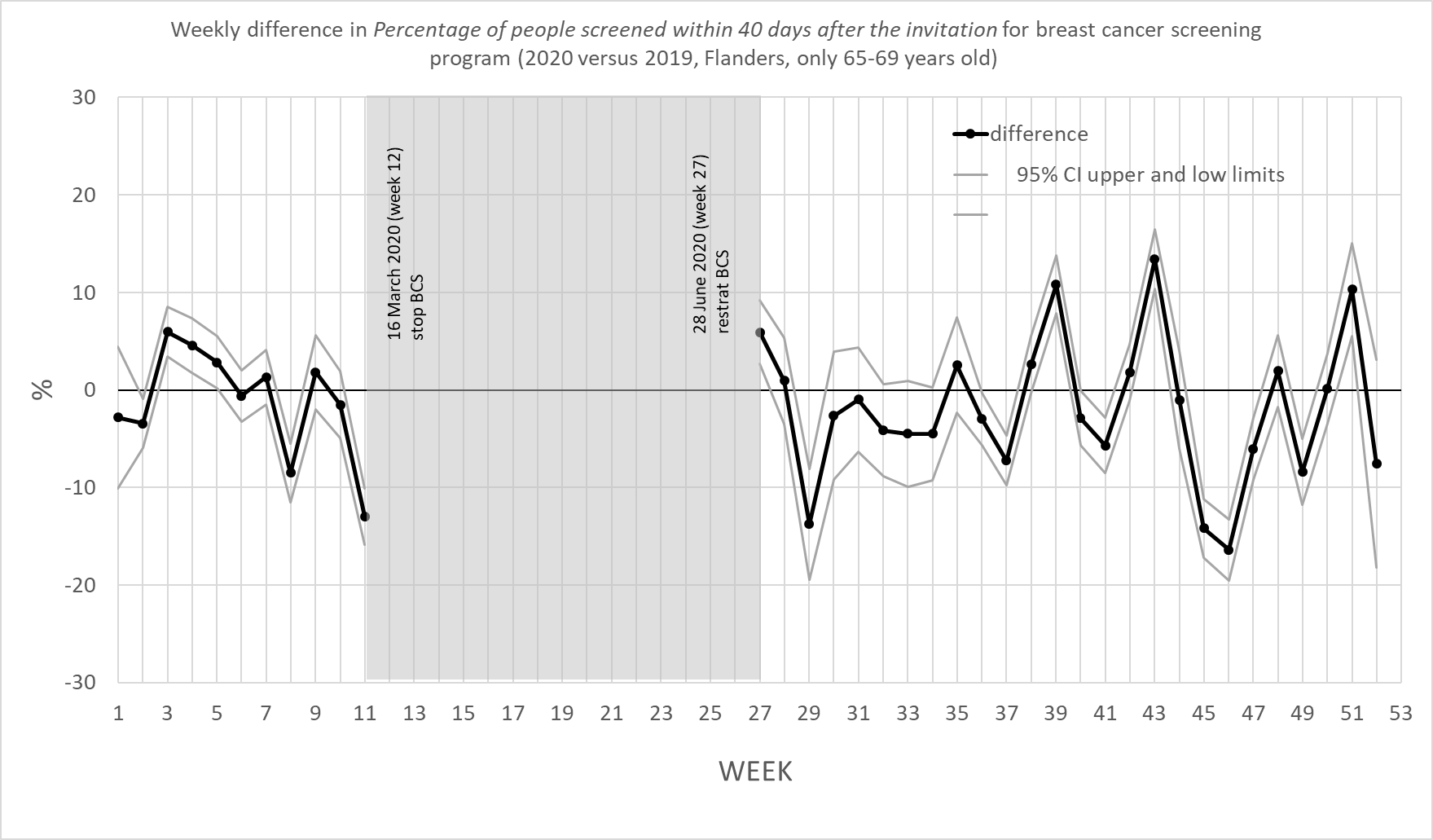 |
| c. 60-64 years old | d. 65-69 years old |

| Figure 2 Weekly difference in percentage of people screened within 40 days after the invitation for breast cancer screening program, by screening history (2020 versus 2019, Flanders). | |
| --- | --- |
| 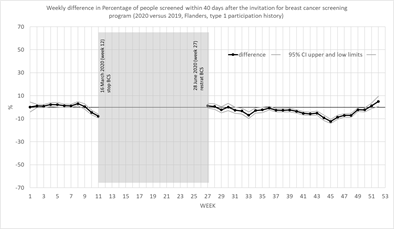 | 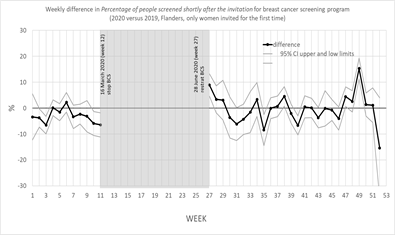 |
| a. type 1 (participated in the last round) | b. type 2 (first-time invitees) |
| 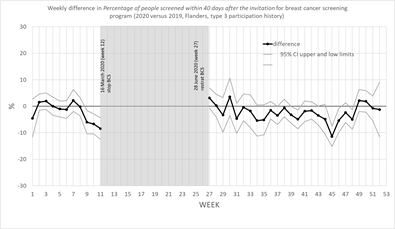 | 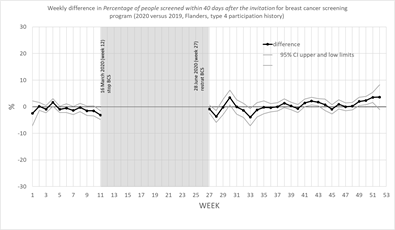 |
| c. type 3 (participated before, but not in the last round) | d. type 4 (never participated) |

| Figure 3 Weekly difference in percentage of people screened within 40 days after the invitation for **colorectal** cancer screening program, by age (2020 versus 2019, Flanders). | |
| --- | --- |
| 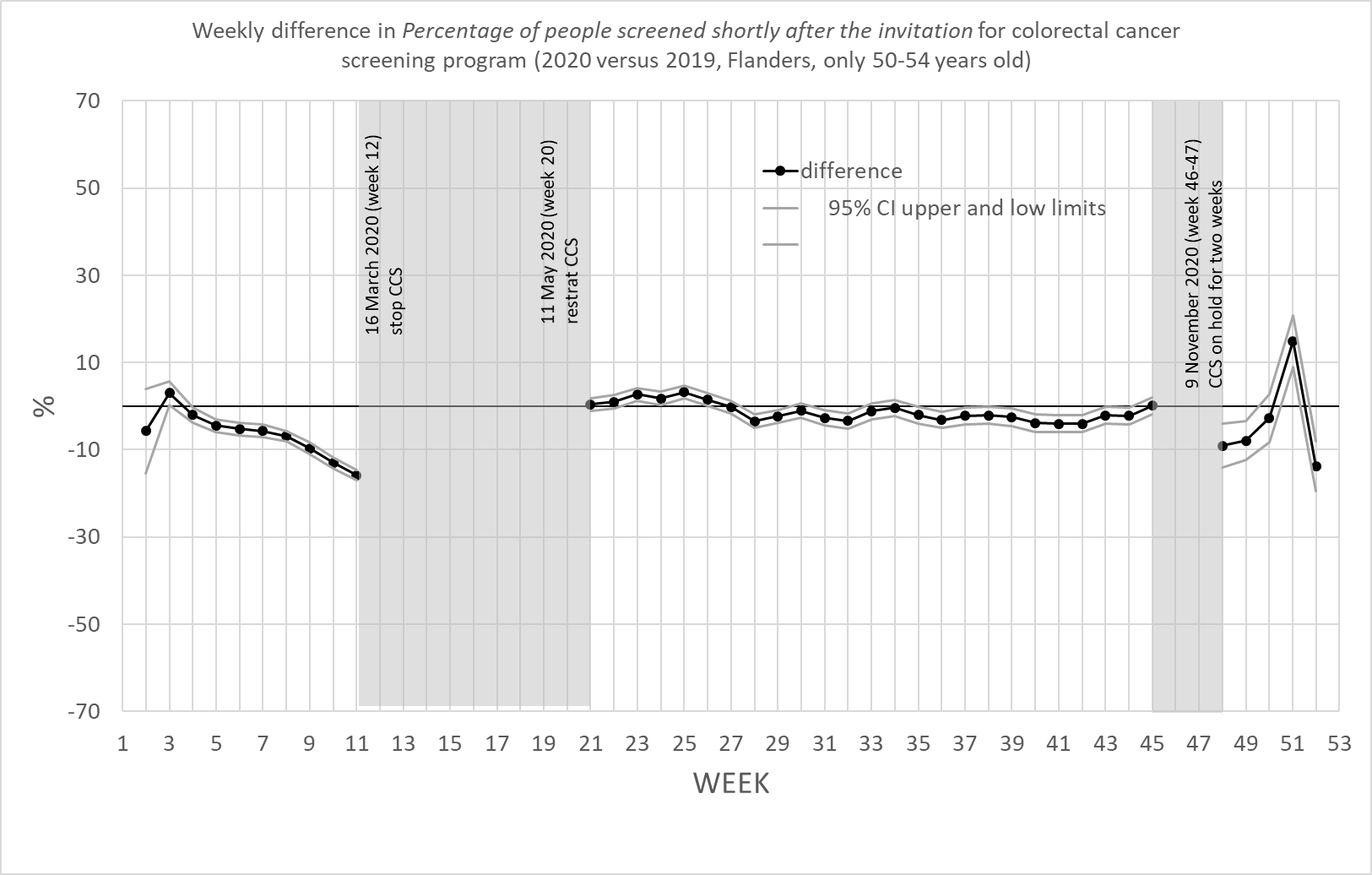 | 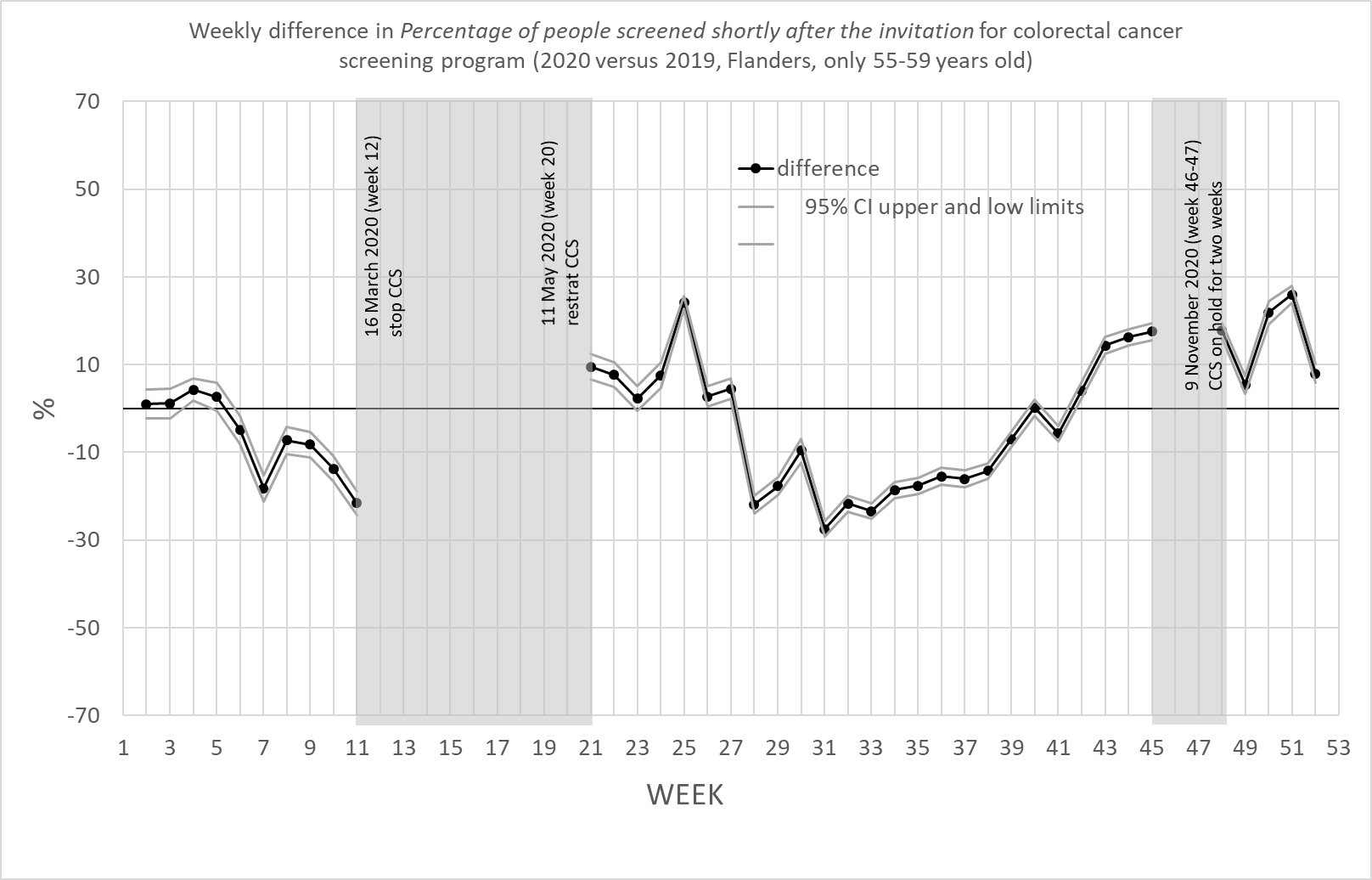 |
| a. 50-54 years old | b. 55-59 years old |
| 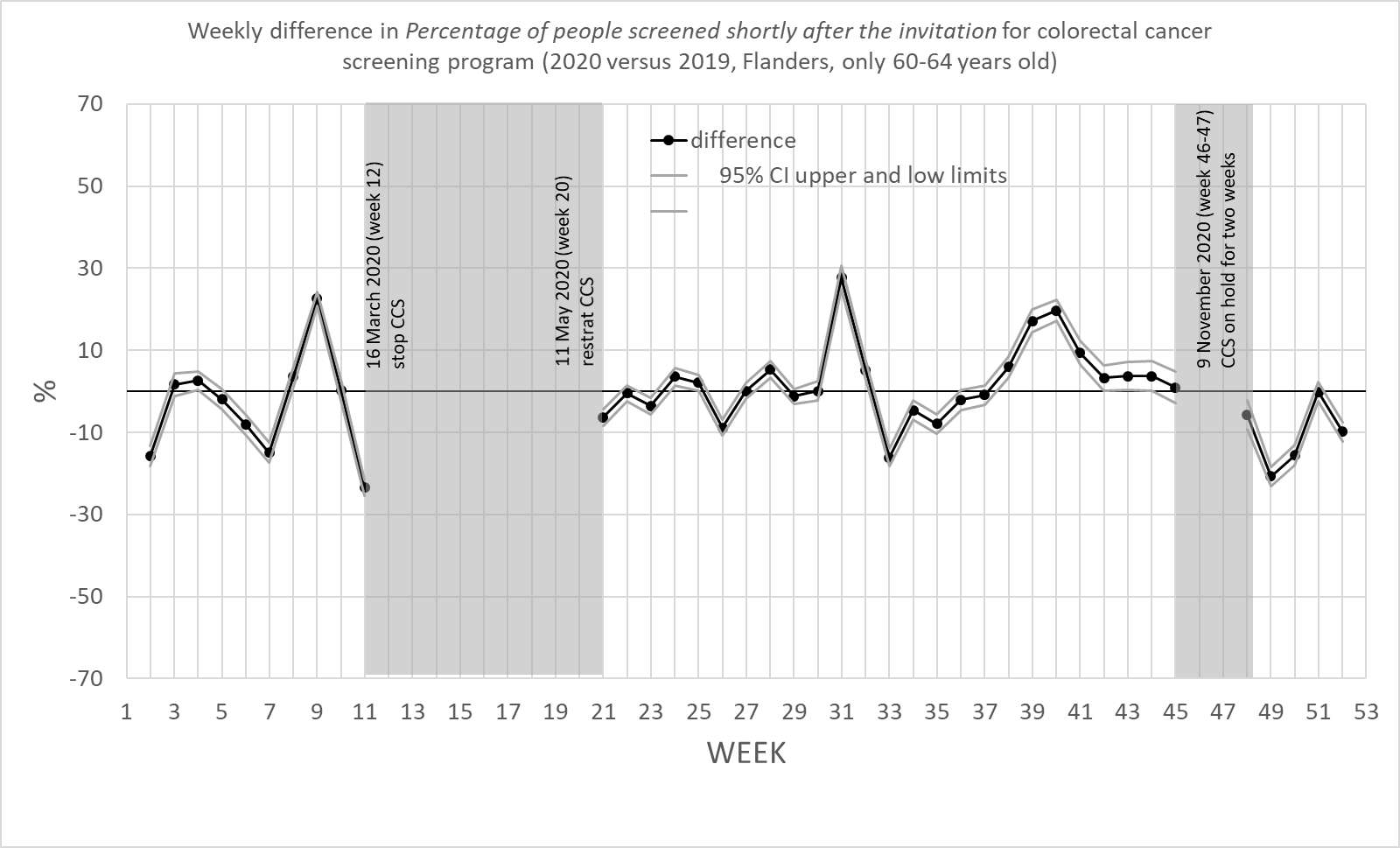 | 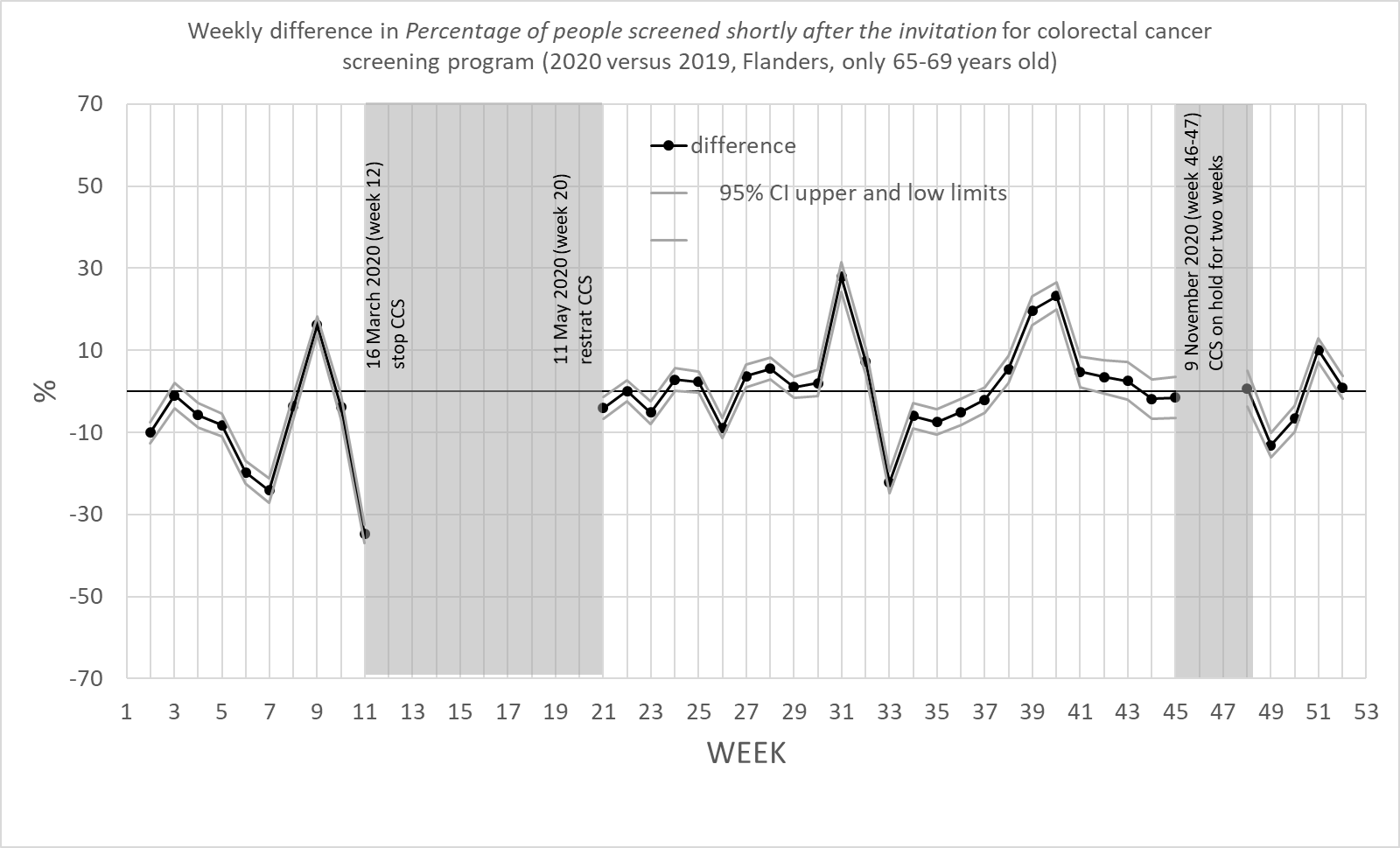 |
| c. 60-64 years old | d. 65-69 years old |
| 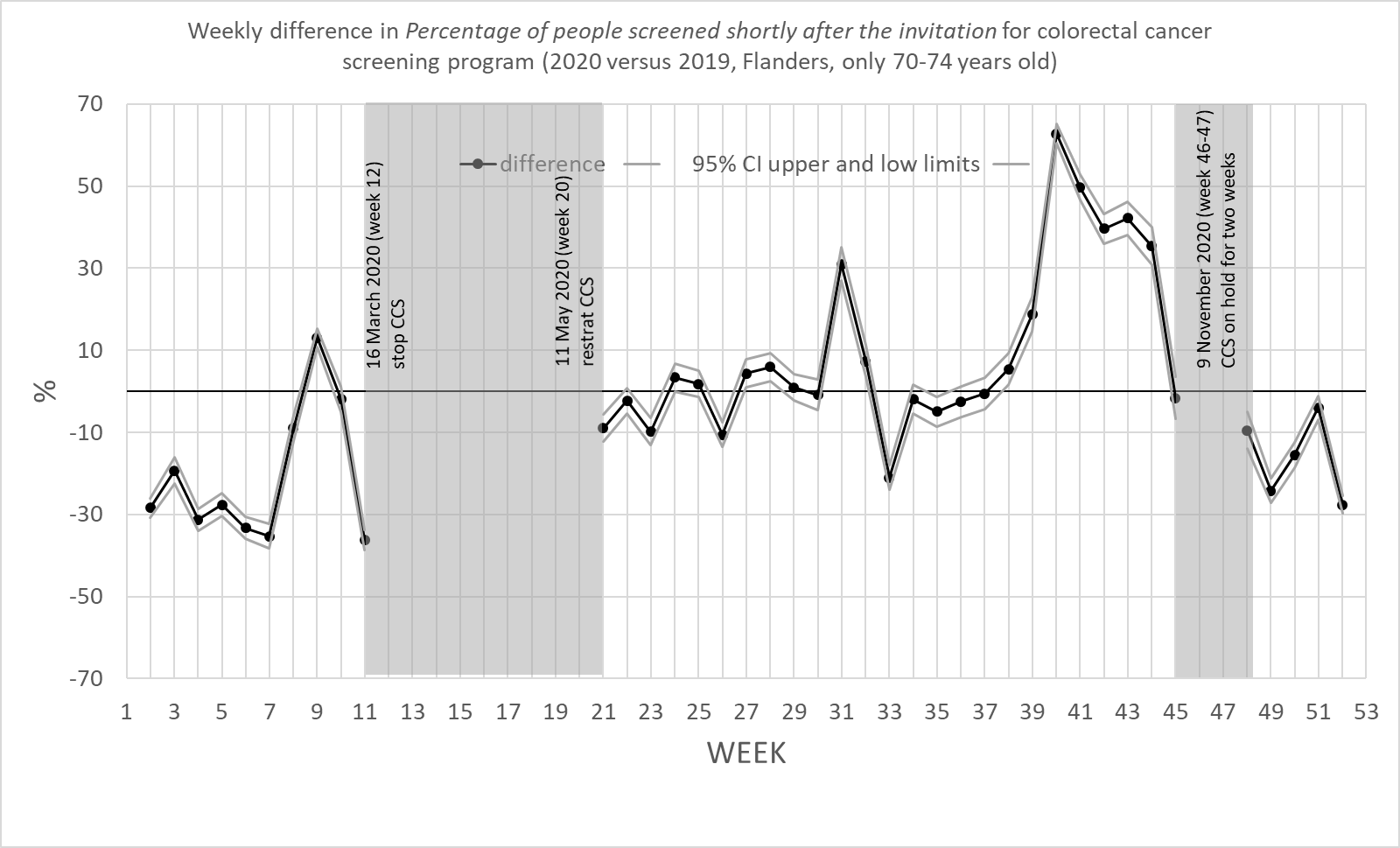 |  |
| e. 70-74 years old |  |

| Figure 4 Weekly difference in percentage of people screened within 40 days after the invitation for colorectal cancer screening program, by screening history (2020 versus 2019, Flanders). | |
| --- | --- |
| 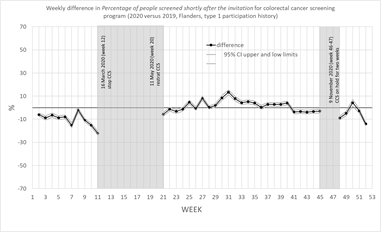 | 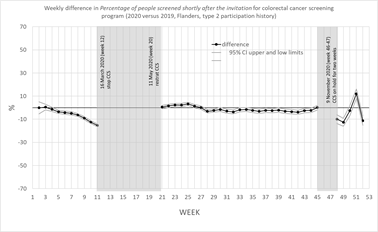 |
| a. type 1 (participated in the last round) | b. type 2 (first-time invitees) |
| 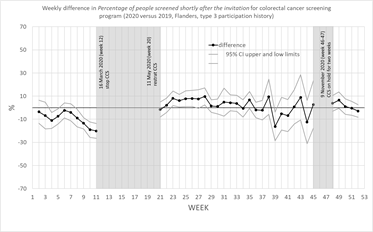 | 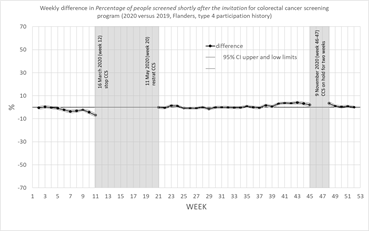 |
| c. type 3 (participated before, but not in the last round) | d. type 4 (never participated) |

| Figure 5 Weekly difference in percentage of people screened within 40 days after the invitation for colorectal cancer screening program, by gender (2020 versus 2019, Flanders). | |
| --- | --- |
| 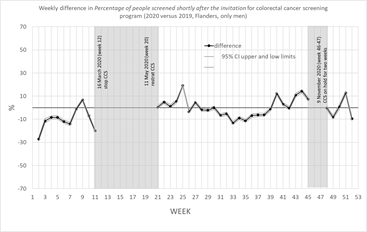 | 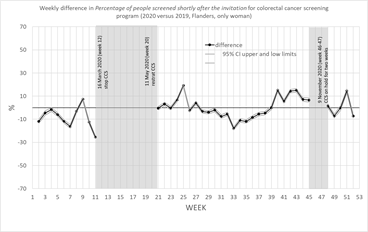 |
| a. men | b. women |

| Figure 6 Weekly difference in mean screening interval for **breast** cancer screening program, by age (2020 versus 2019, Flanders). | |
| --- | --- |
| 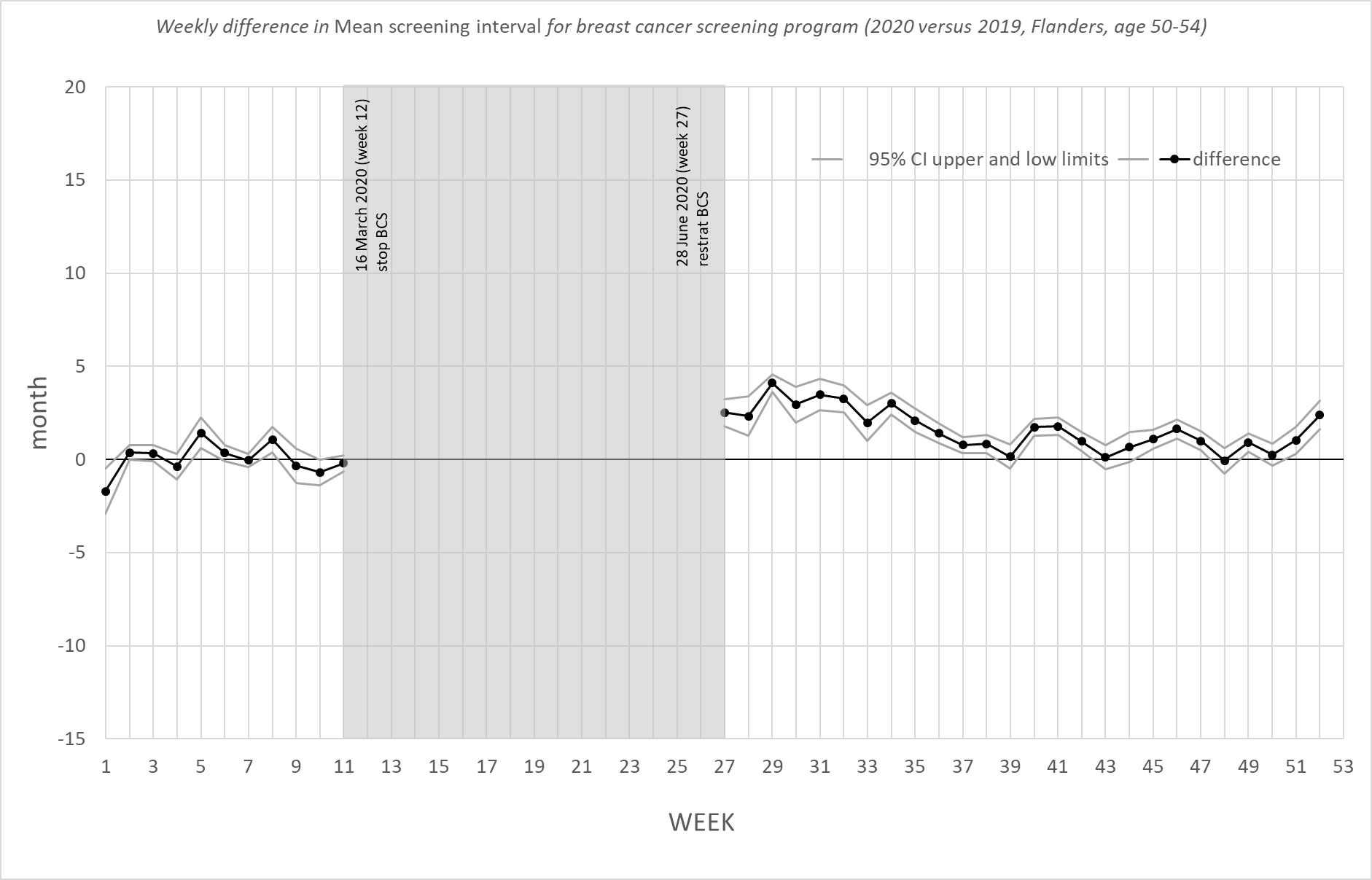 | 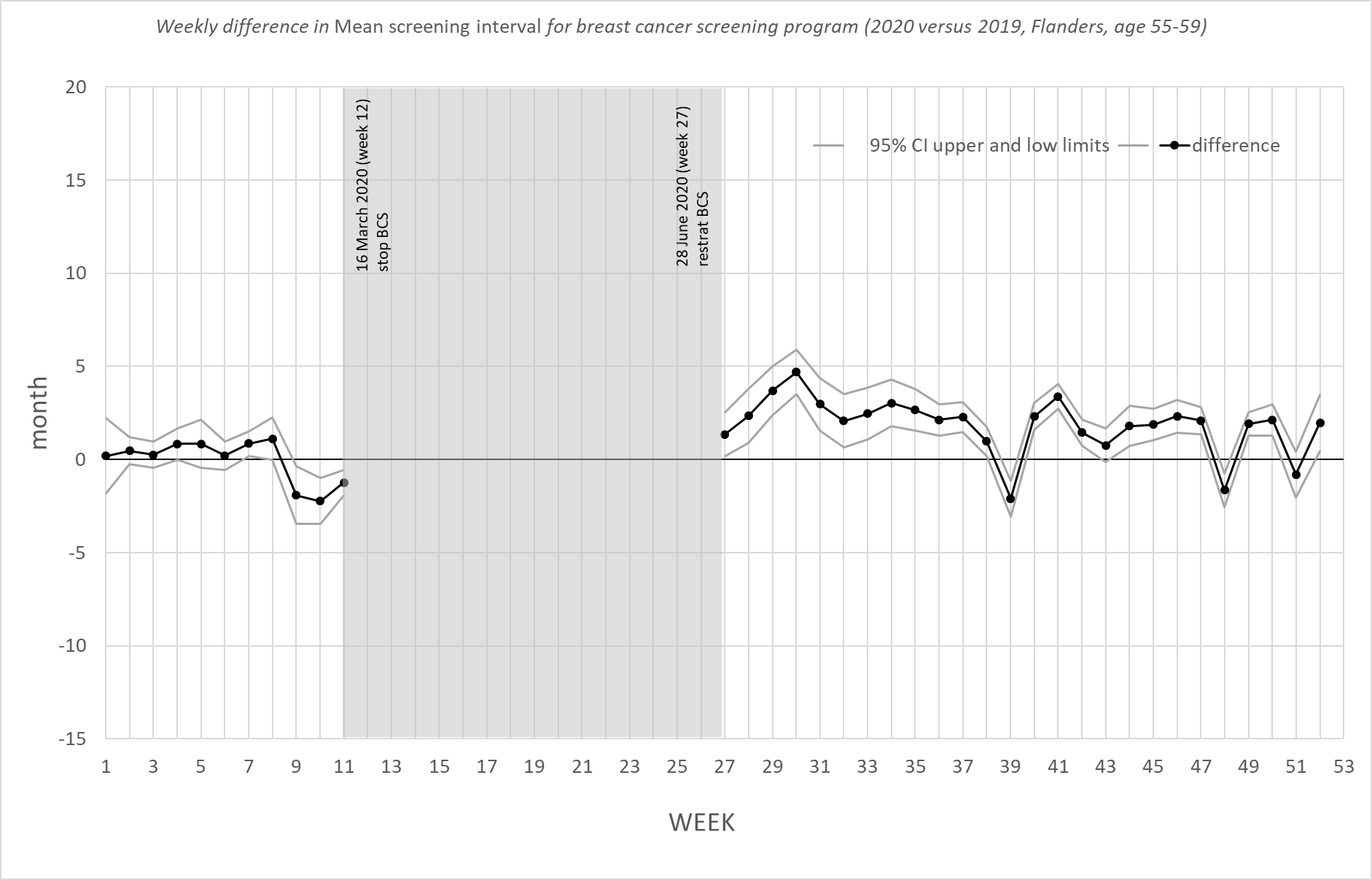 |
| a. 50-54 years old | b. 55-59 years old |
| 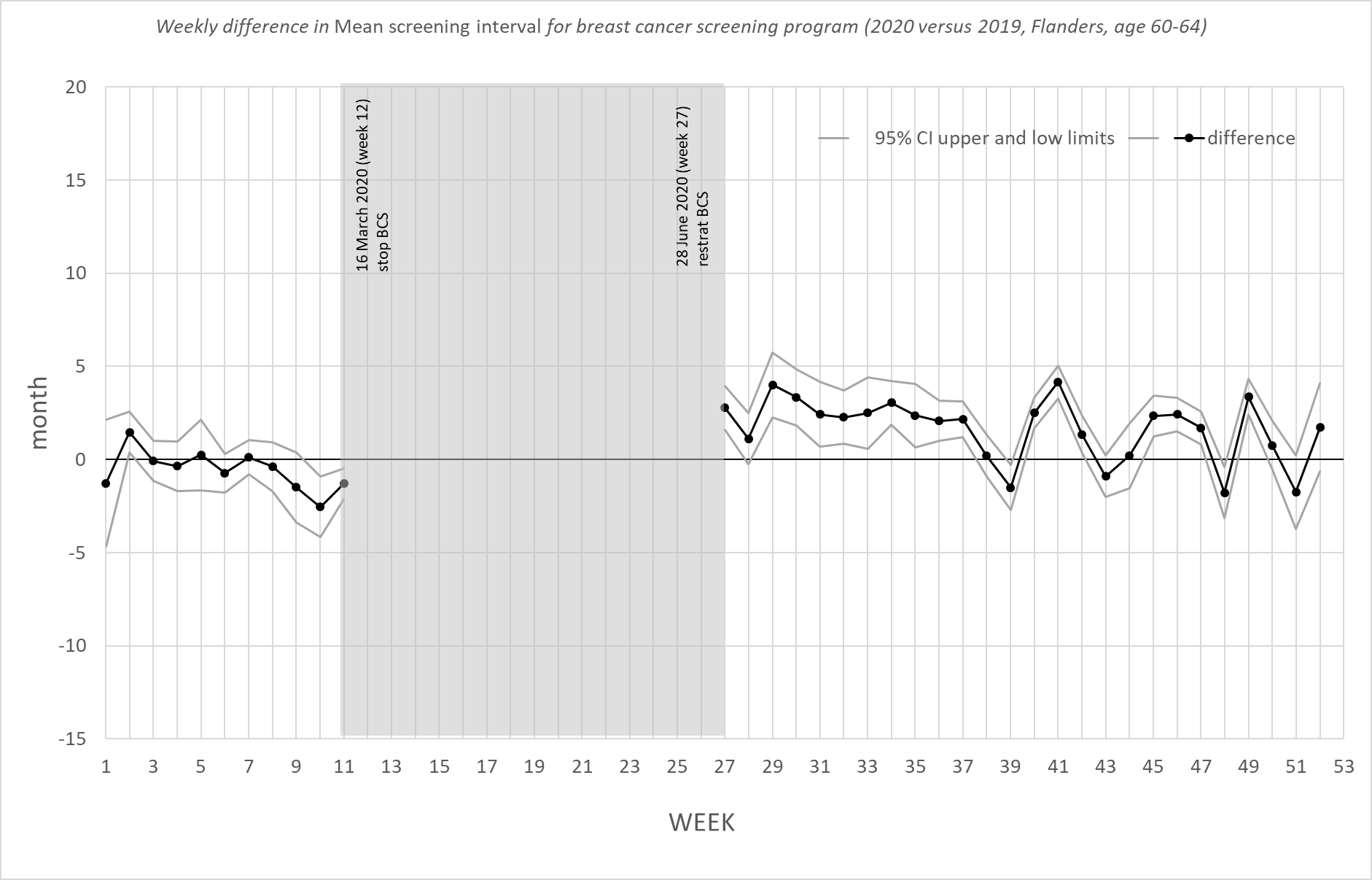 | 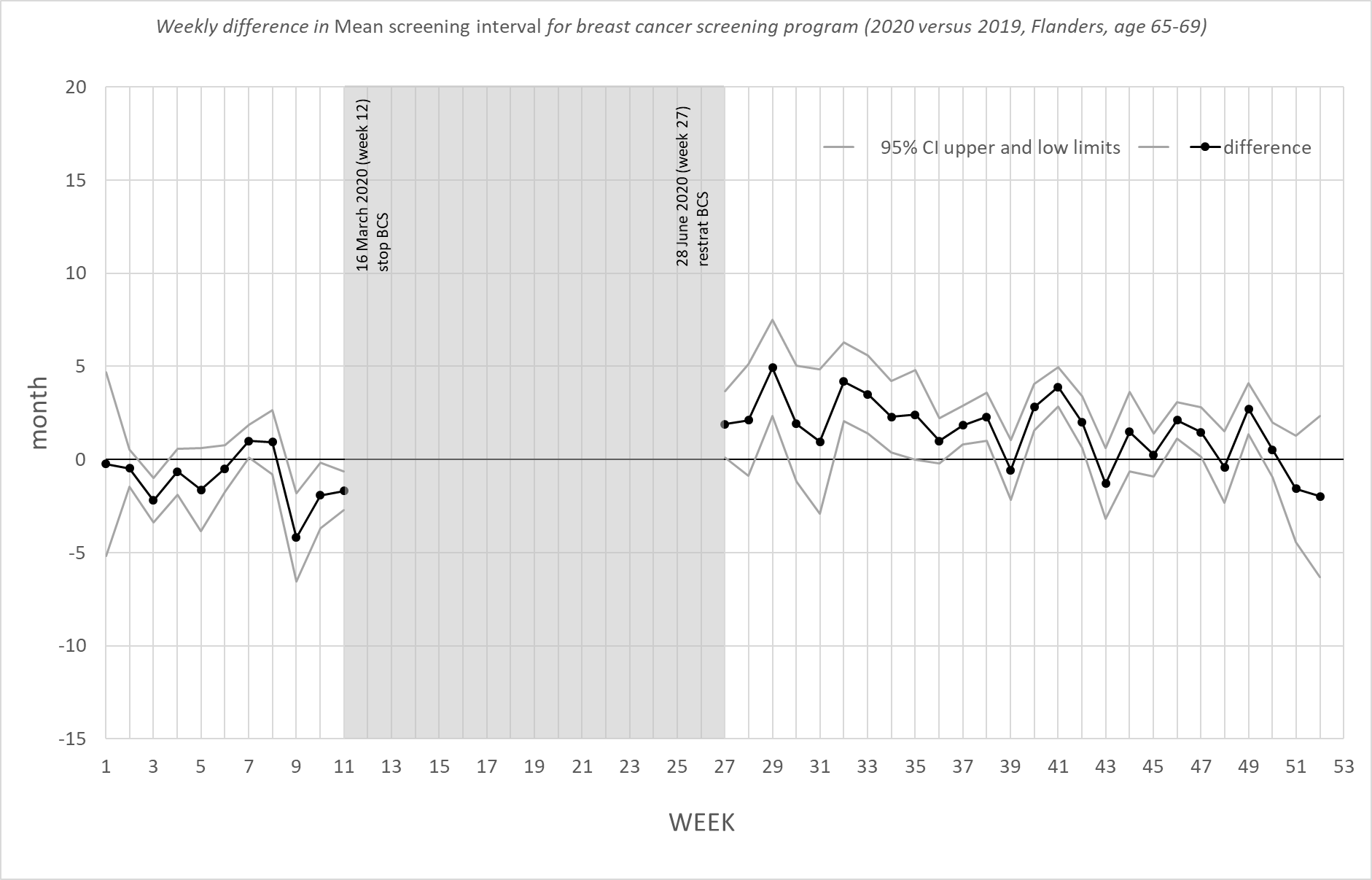 |
| c. 60-64 years old | d. 65-69 years old |

| Figure 7 Weekly difference in mean screening interval for **breast** cancer screening program, by screening history (2020 versus 2019, Flanders). | |
| --- | --- |
| 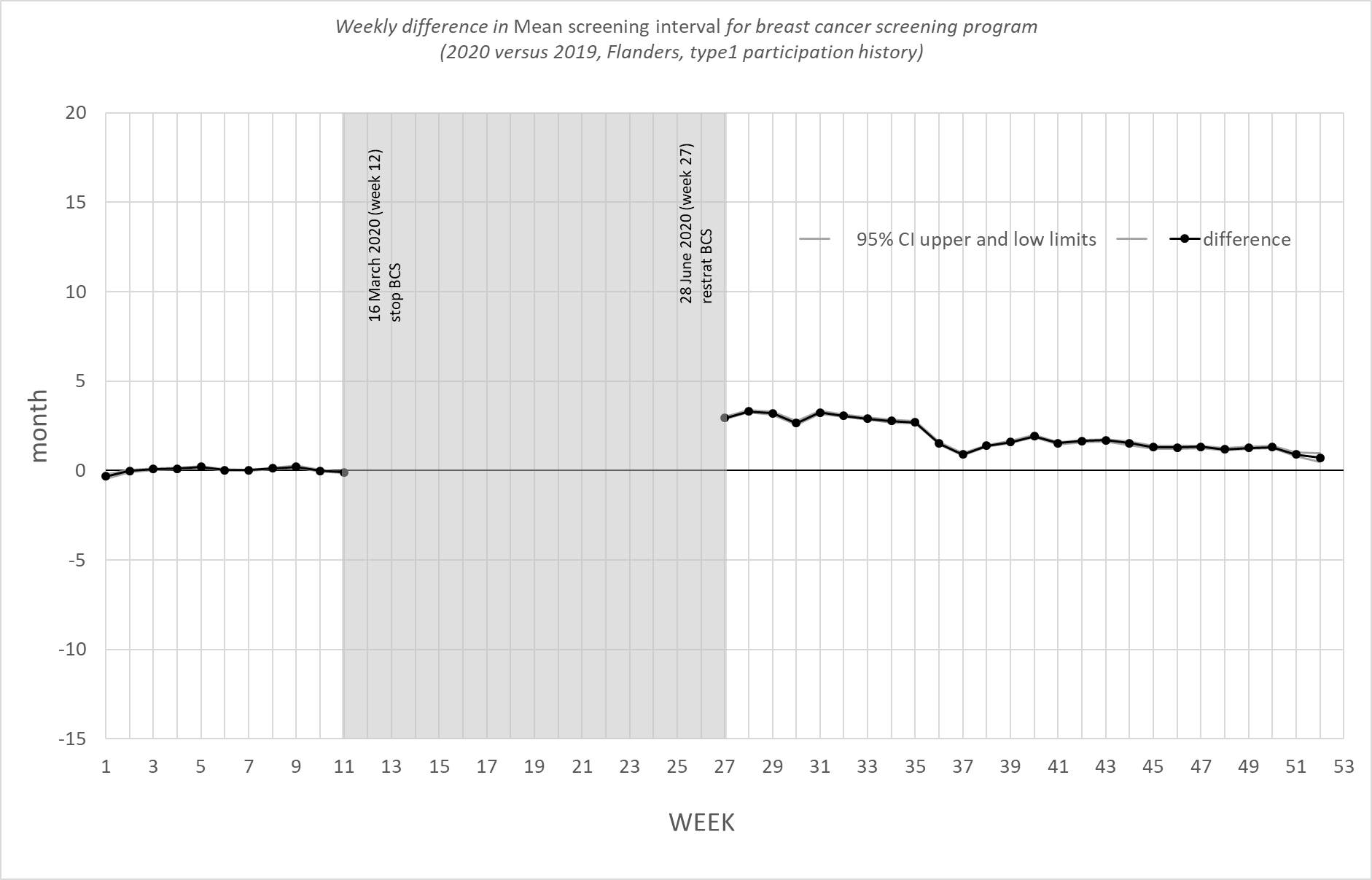 | 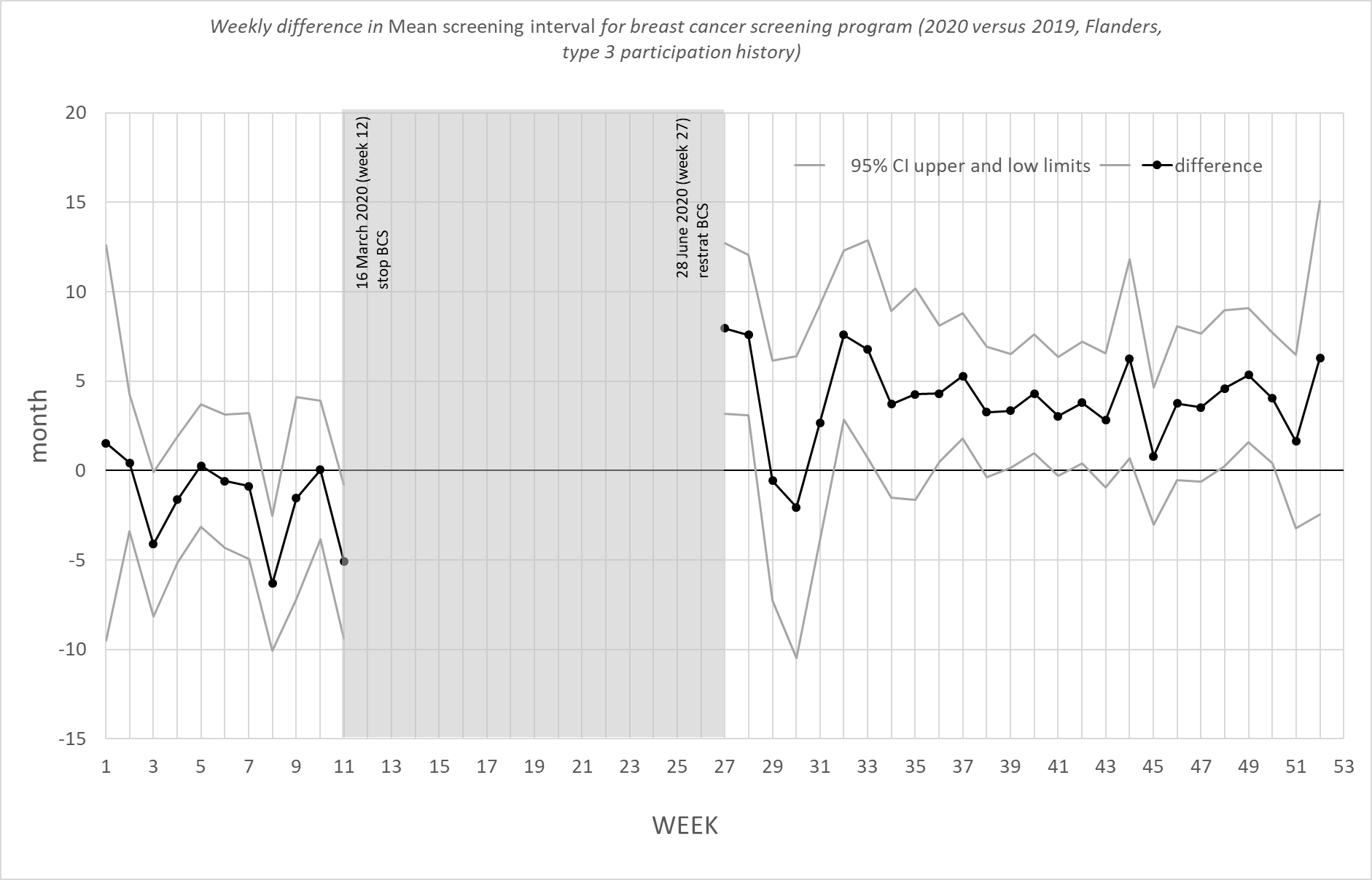 |
| a. type 1 (participated in the last round) | b. type 3 (participated before, but not in the last round) |

| Figure 8 Weekly difference in mean screening interval for **colorectal** cancer screening program, stratified by age (2020 versus 2019, Flanders). | |
| --- | --- |
| 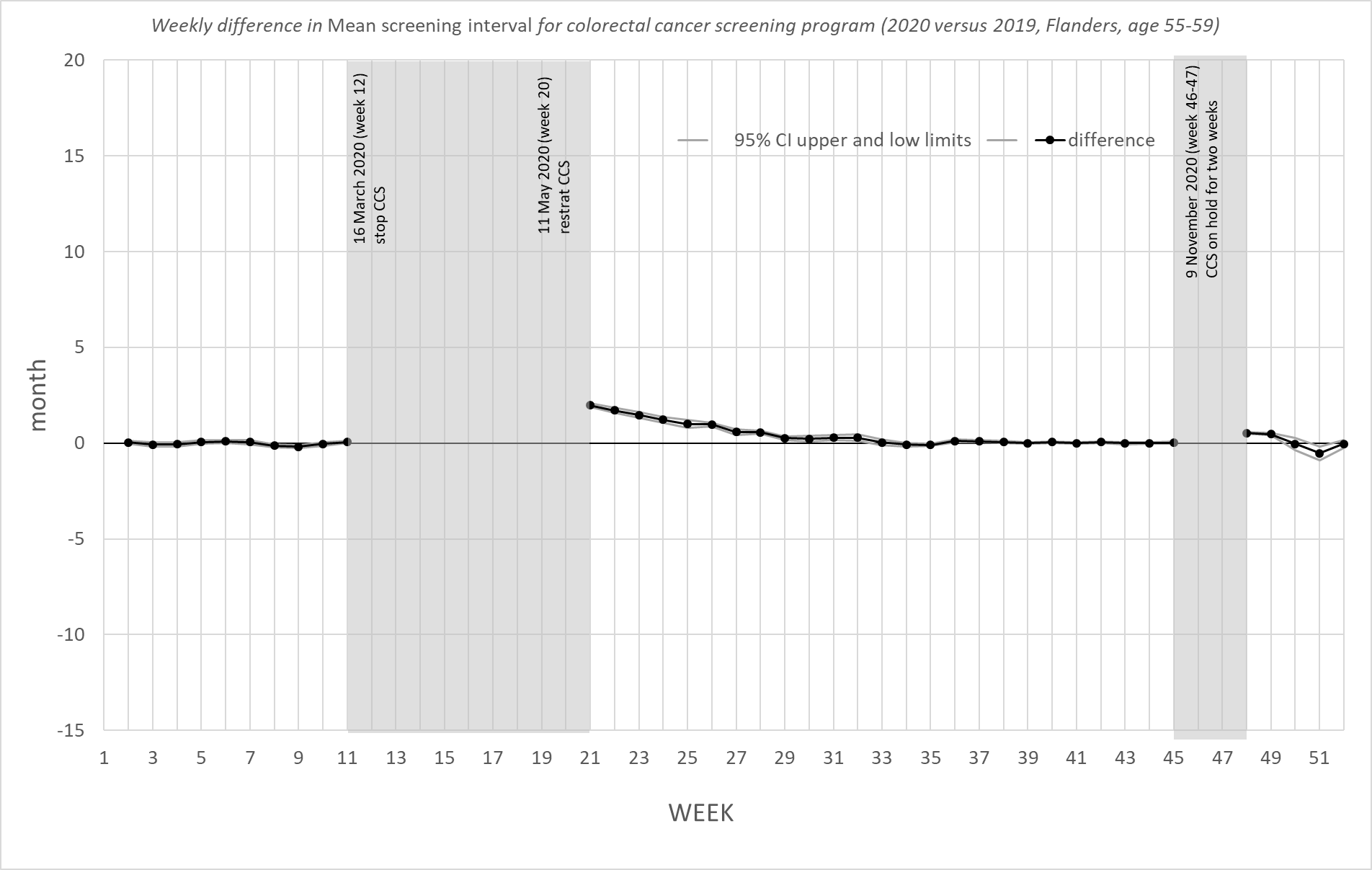 | 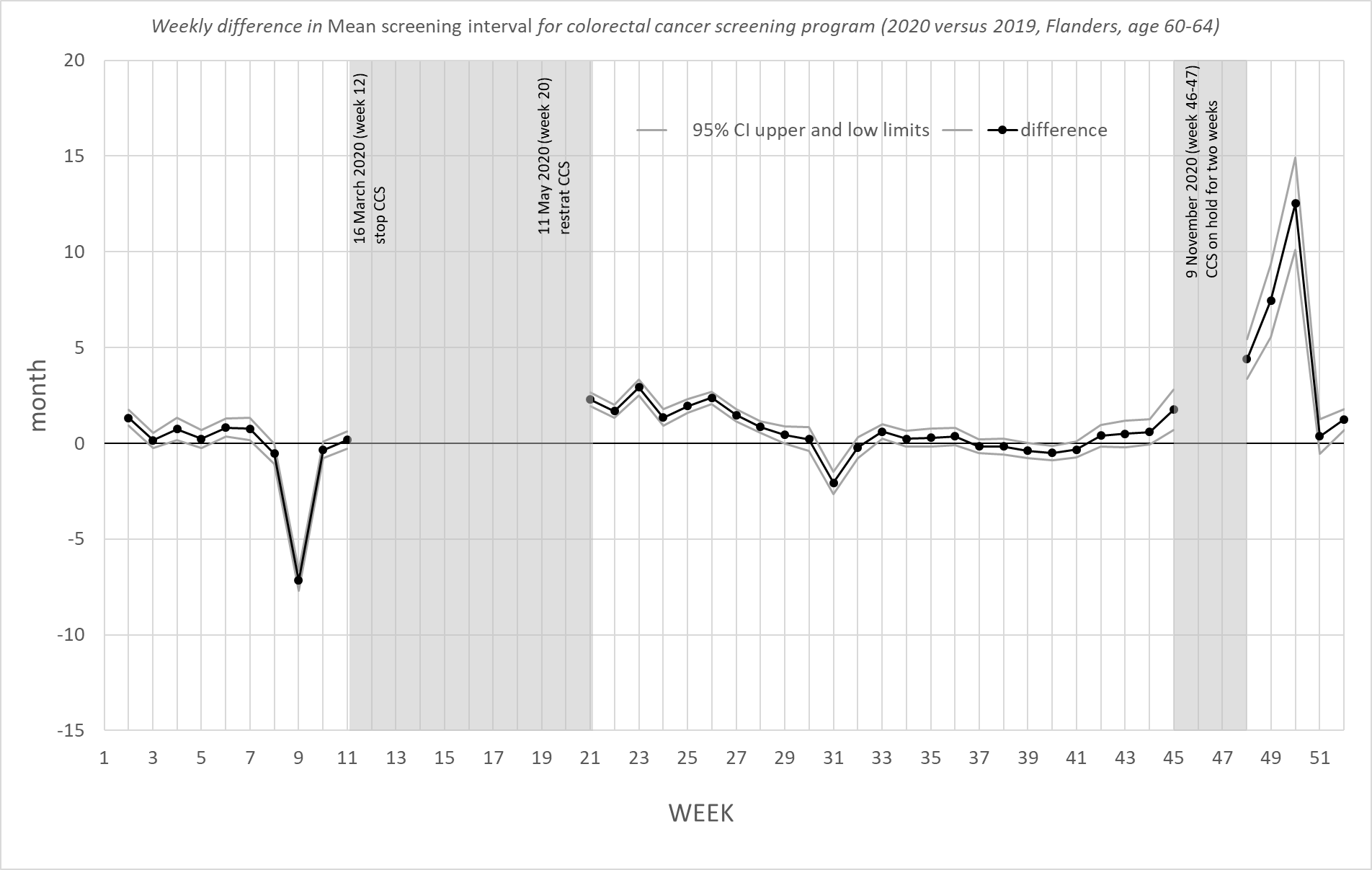 |
| a. 55-59 years old | b. 60-64 years old |
| 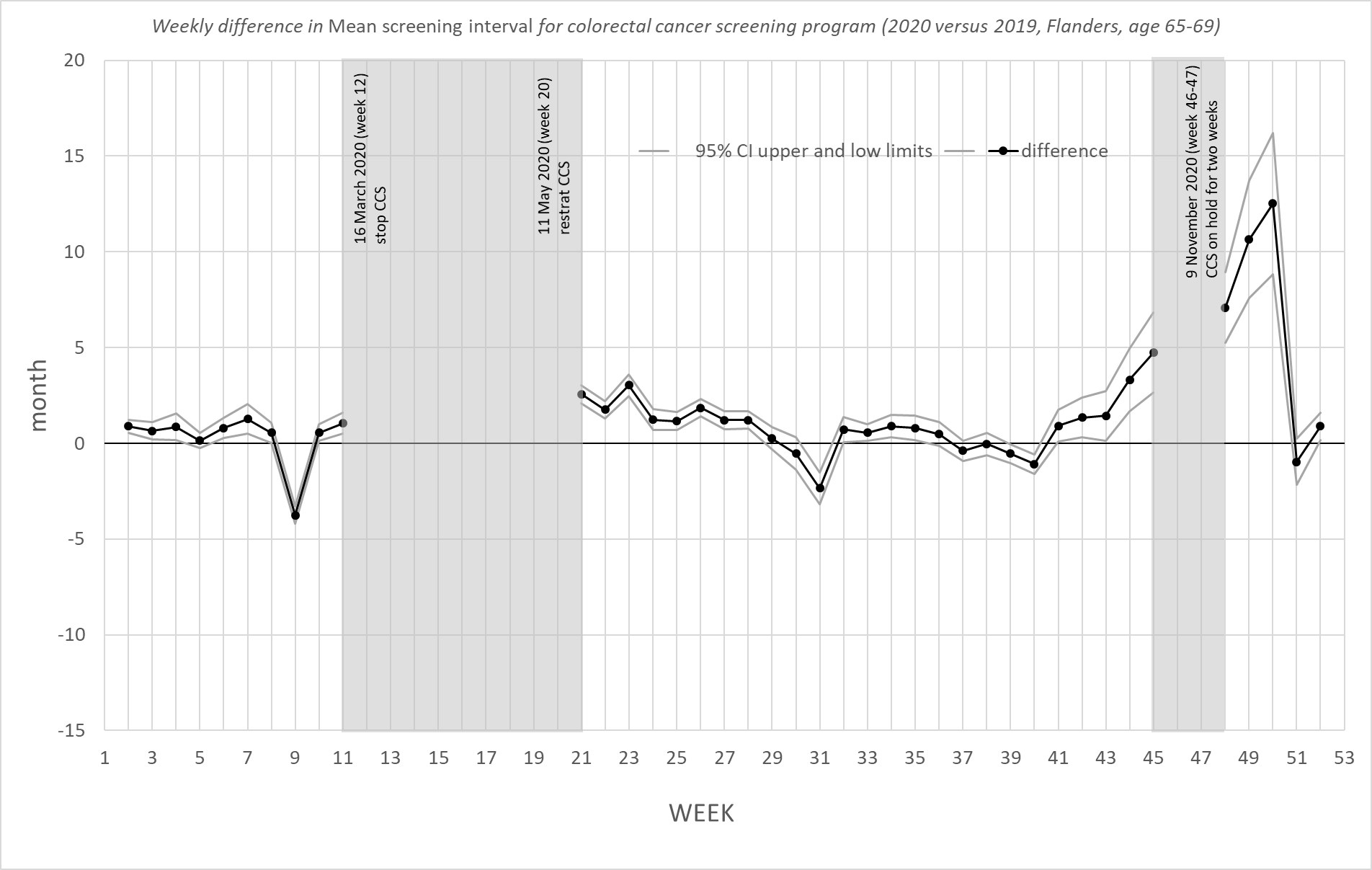 | 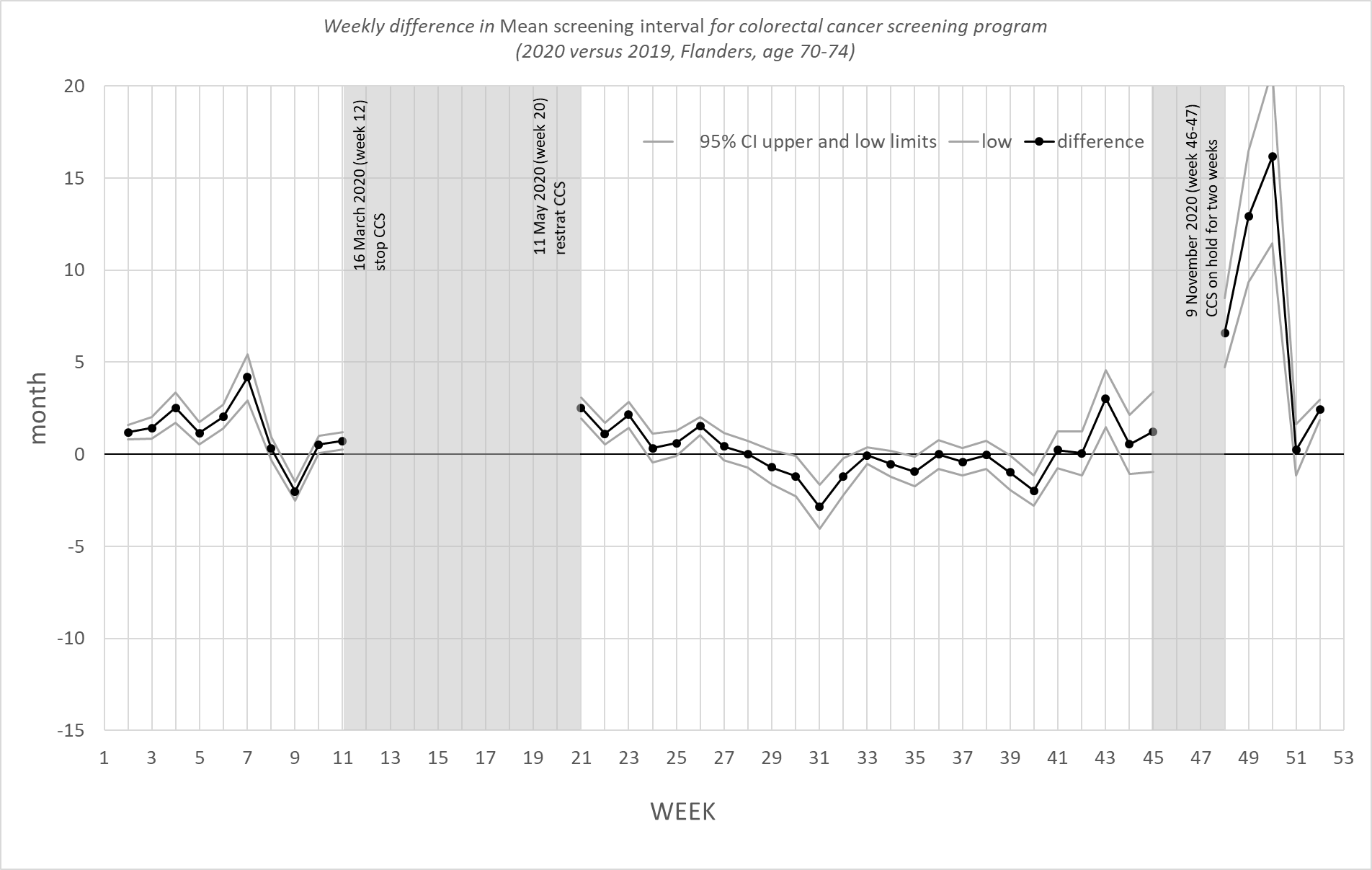 |
| c. 65-69 years old | d. 70-74 years old |

| Figure 9 Weekly difference in mean screening interval for **colorectal** cancer screening program, by screening history (2020 versus 2019, Flanders). | | | |
| --- | --- | --- | --- |
| 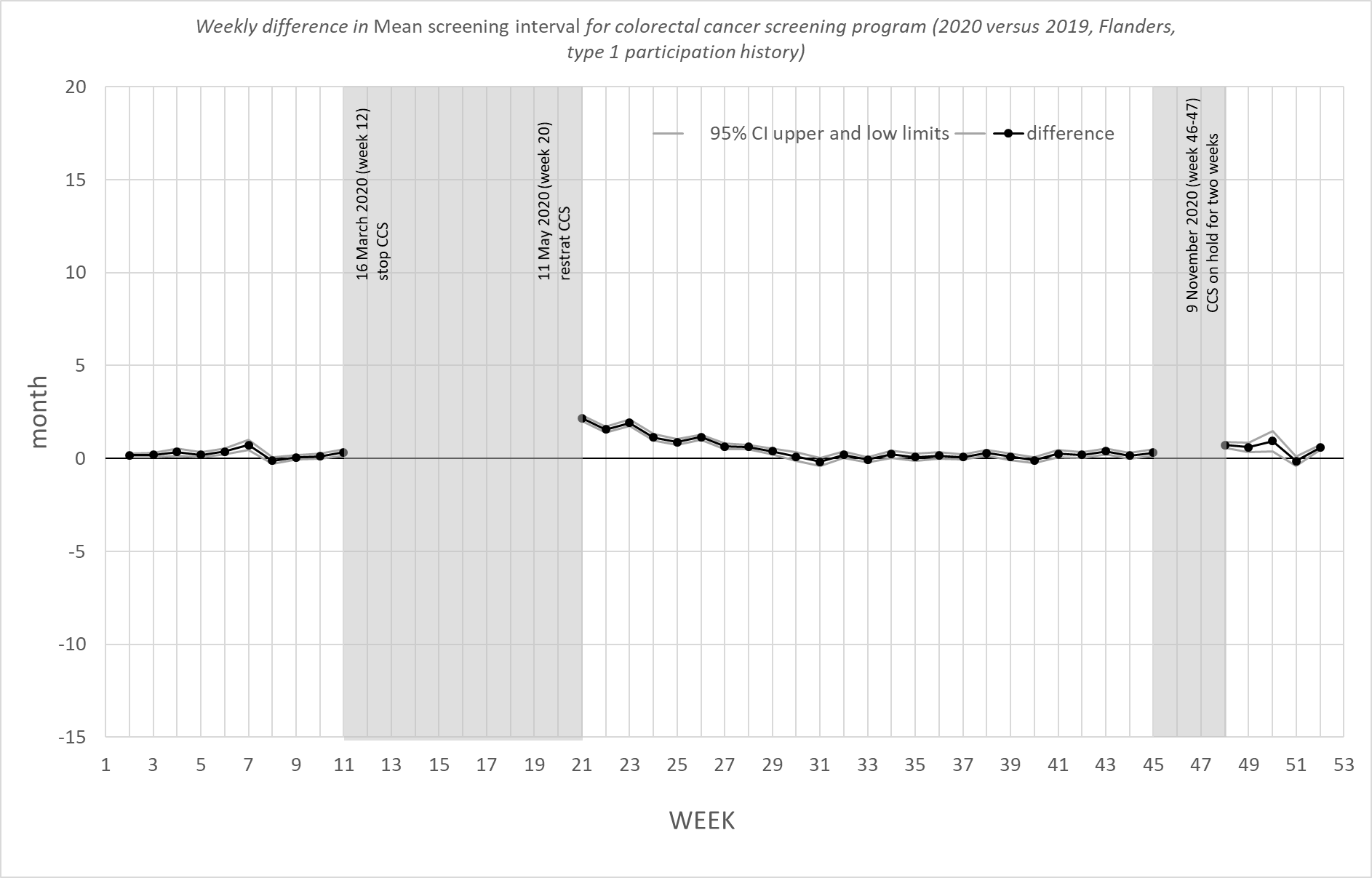 | | 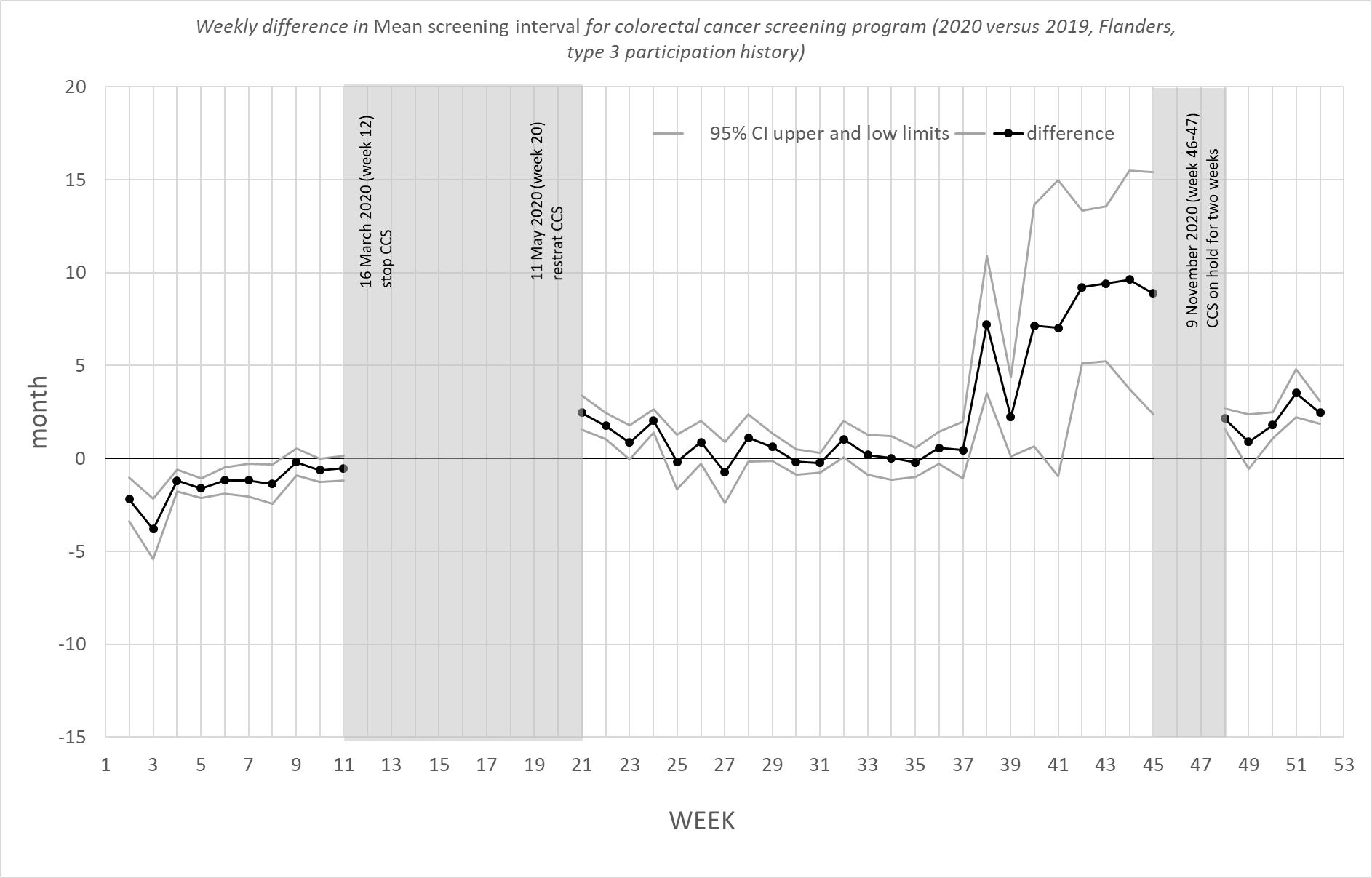 | |
| a. type 1 (participated in the last round ) | | b. type 3 (participated before, but not in the last round) | |
|  | | |  |
| Figure 10 Weekly difference in mean screening interval for **colorectal** cancer screening program, by gender (2020 versus 2019, Flanders). | | |  |
| 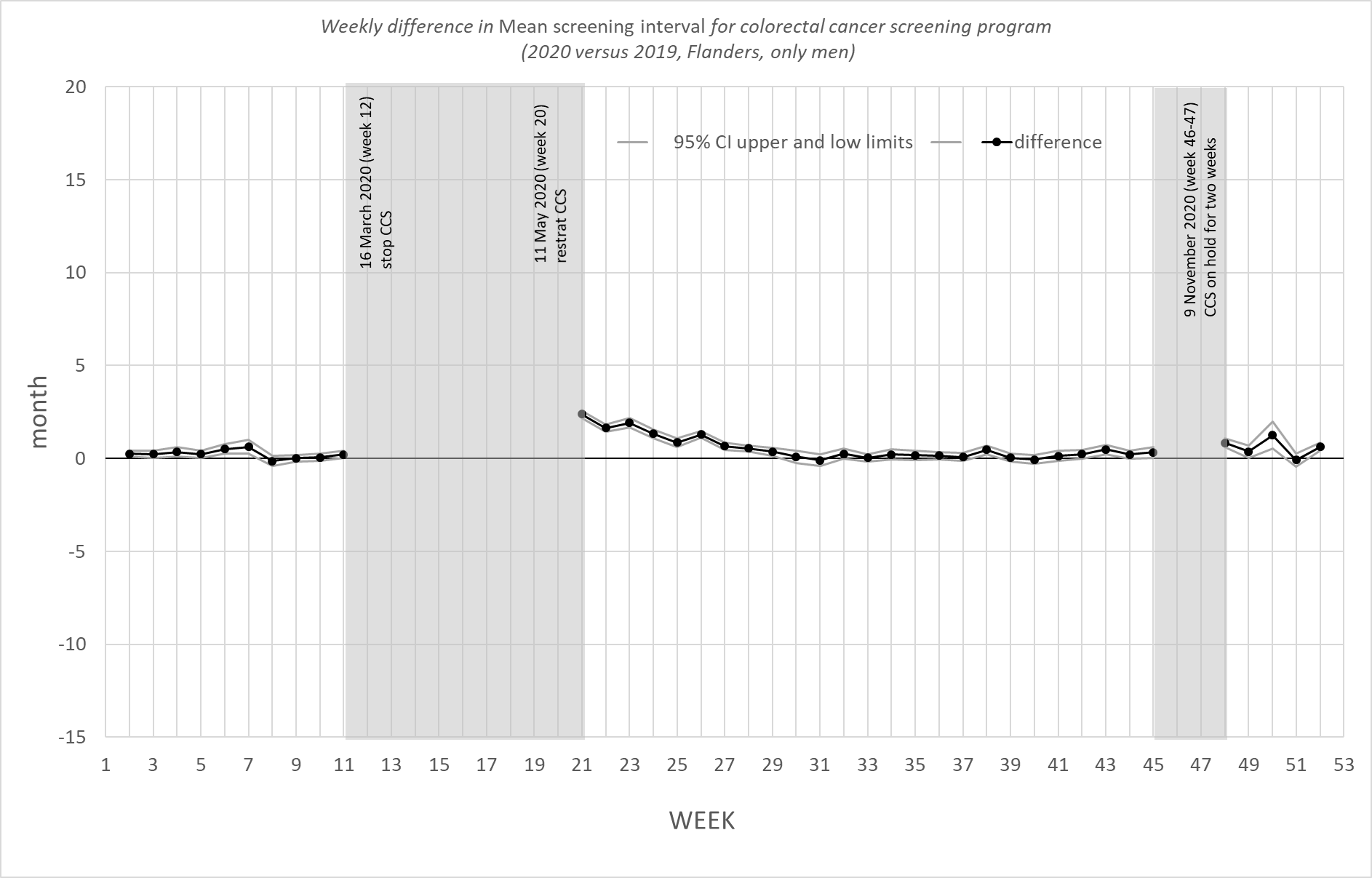 | 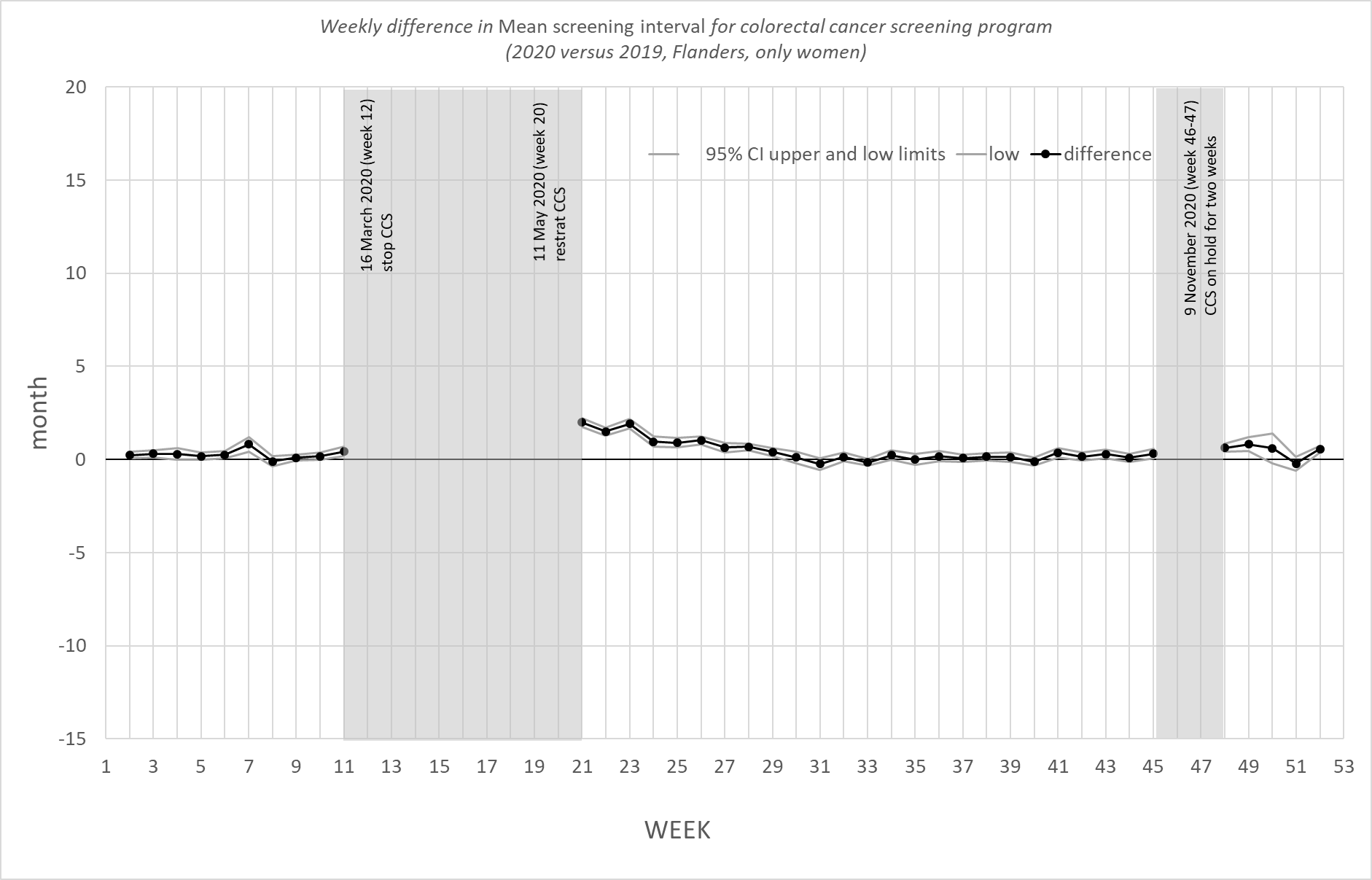 | |  |
| a. men | b. women | |  |

| Figure 11 Weekly difference in mean screening interval for **cervical** cancer screening program, stratified by age (2020 versus 2019, Flanders). | |
| --- | --- |
| 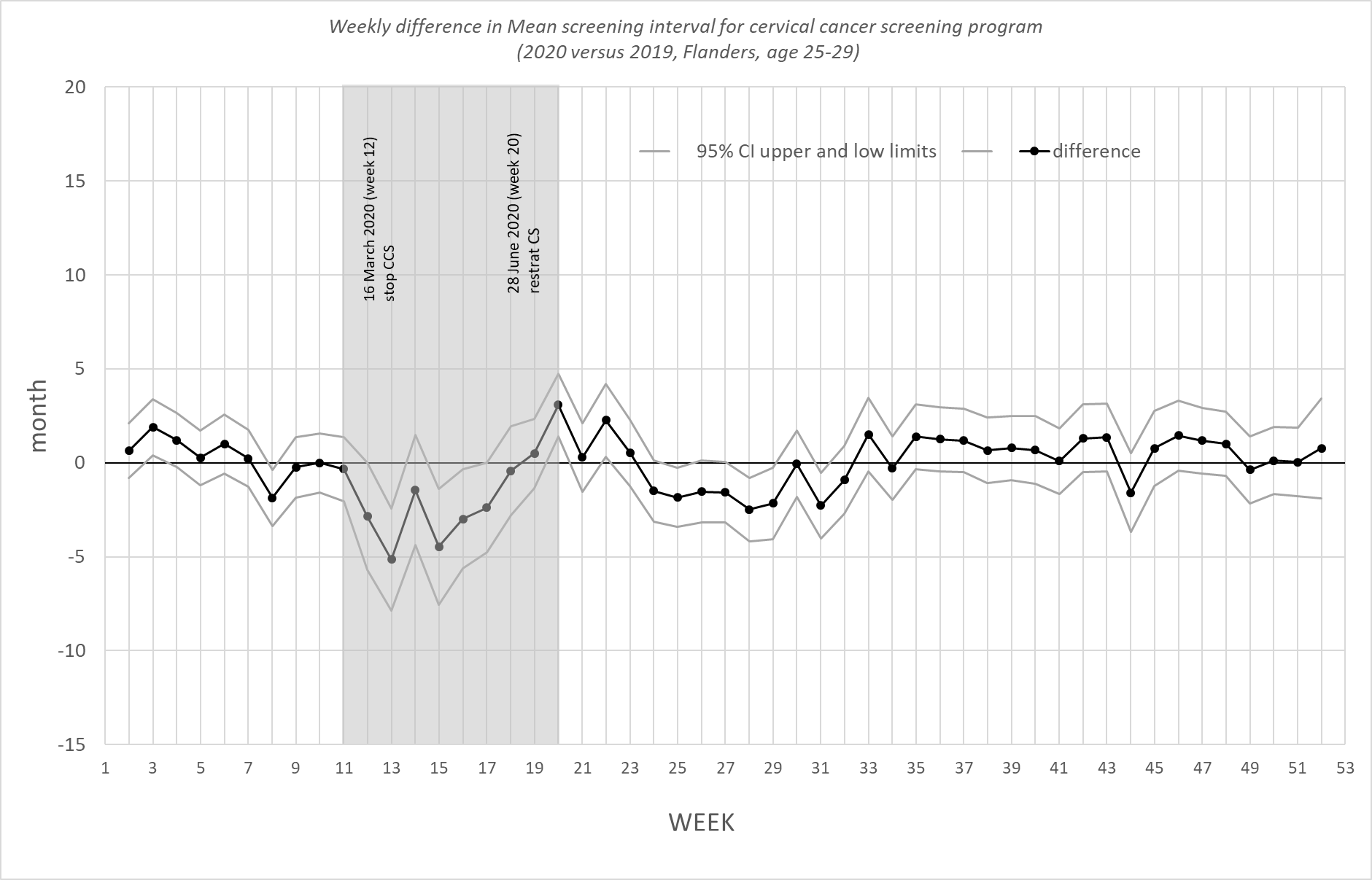 | 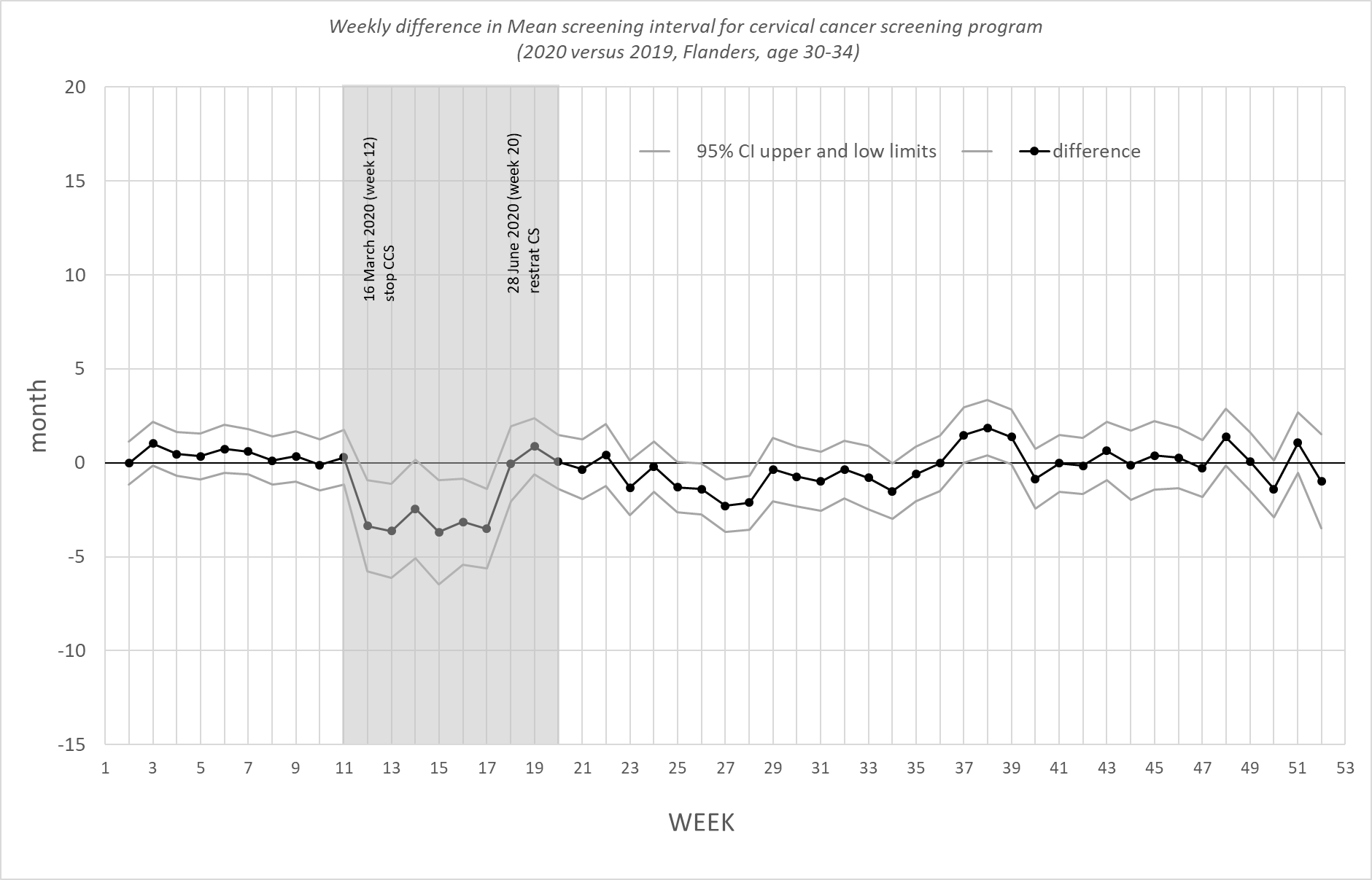 |
| a. 25-29 years old | b. 30-34 years old |
| 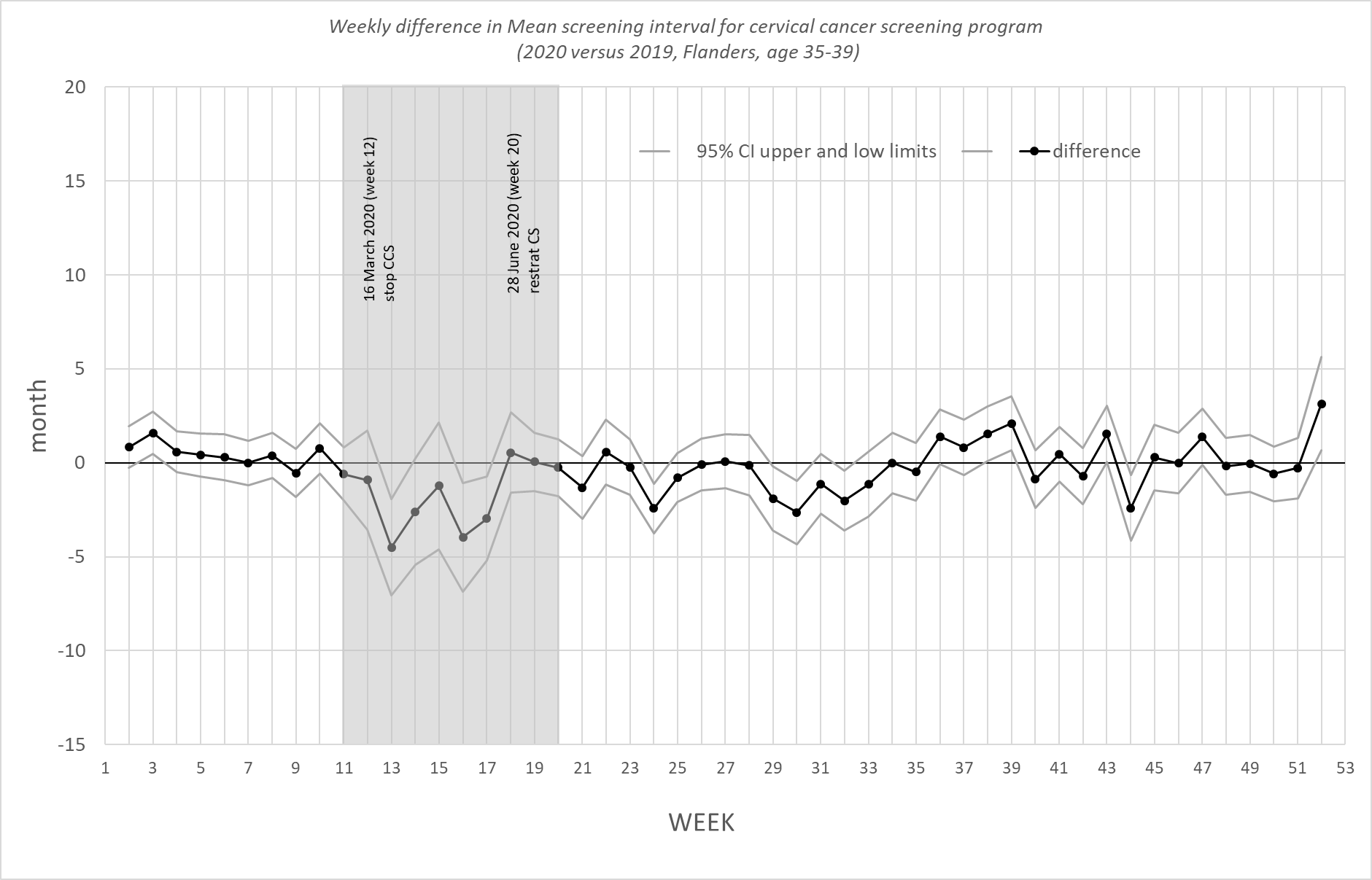 | 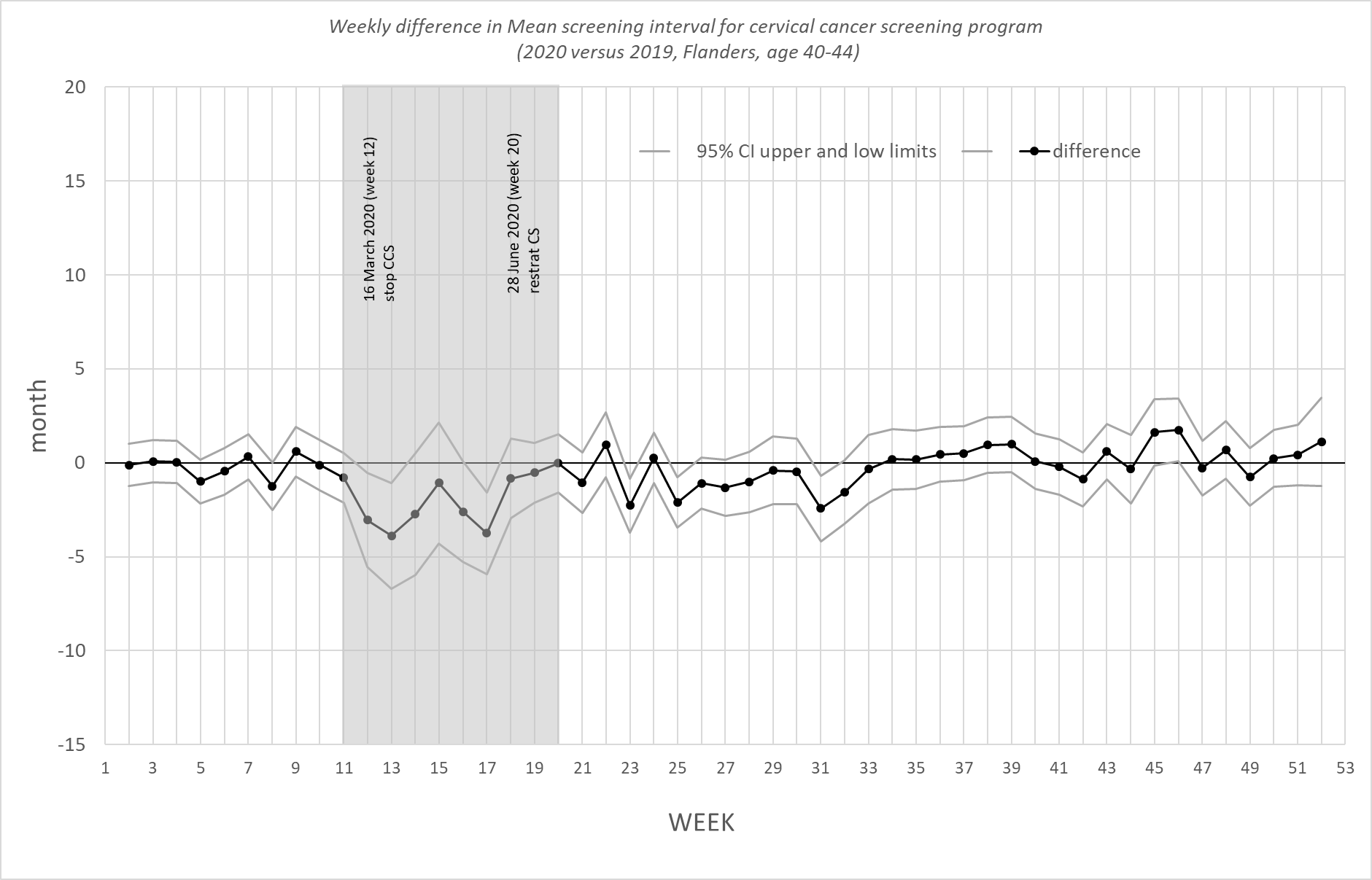 |
| c. 35-39 years old | d. 40-44 years old |

| Figure 12 Weekly difference in mean screening interval for **cervical** cancer screening program, stratified by age (2020 versus 2019, Flanders). | |
| --- | --- |
| 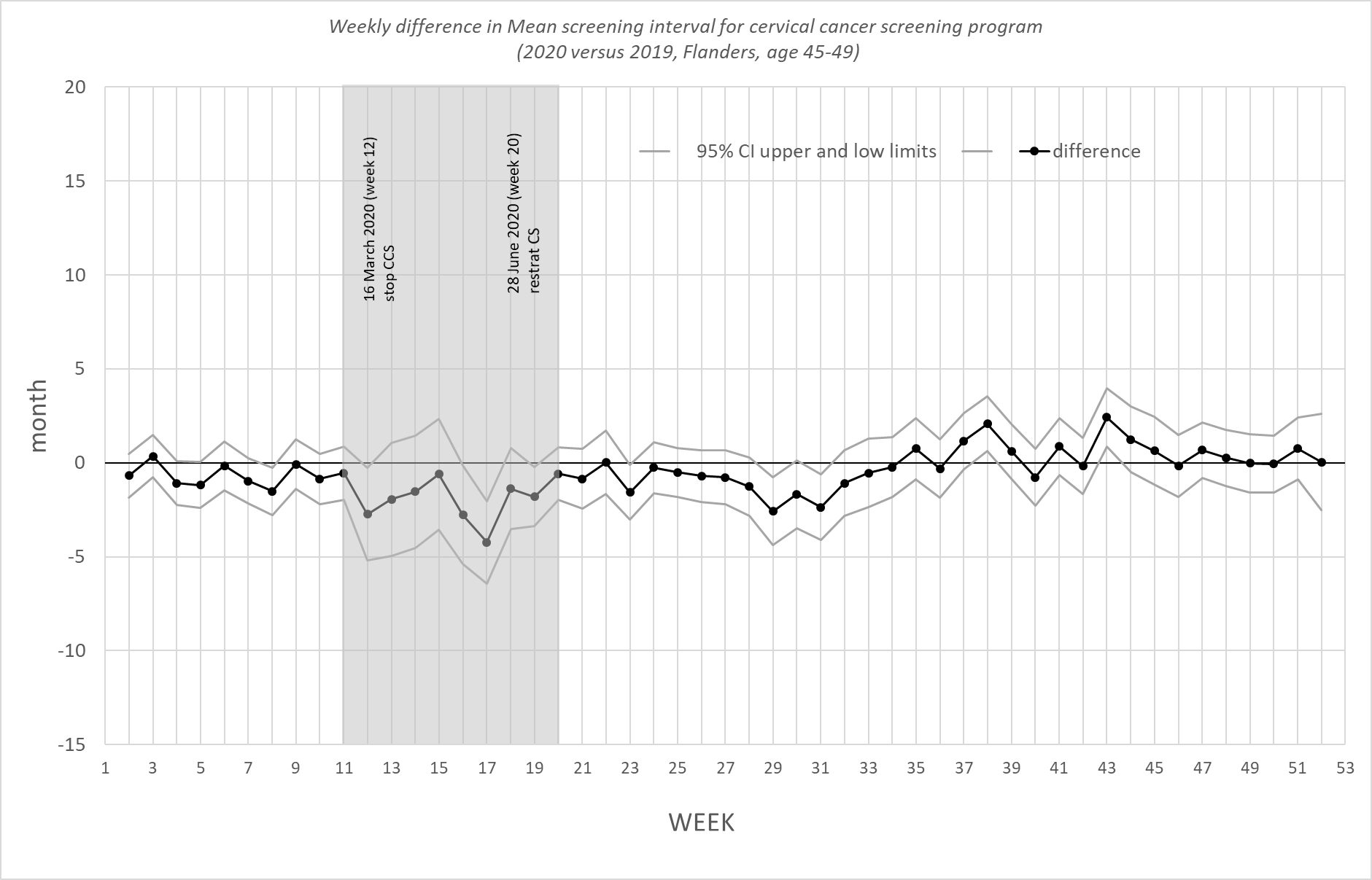 | 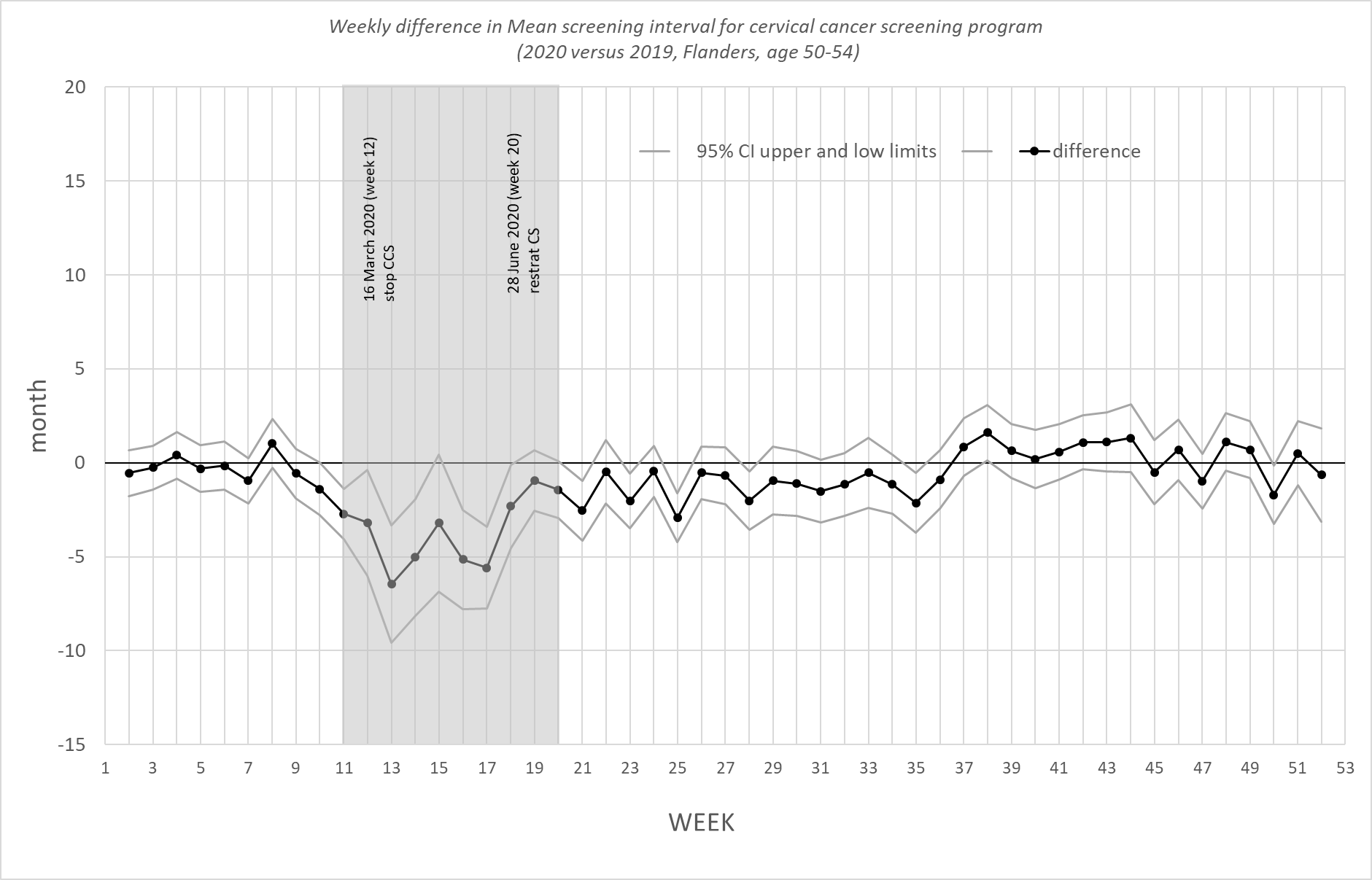 |
| e. 45-49 years old | f. 50-54 years old |
| 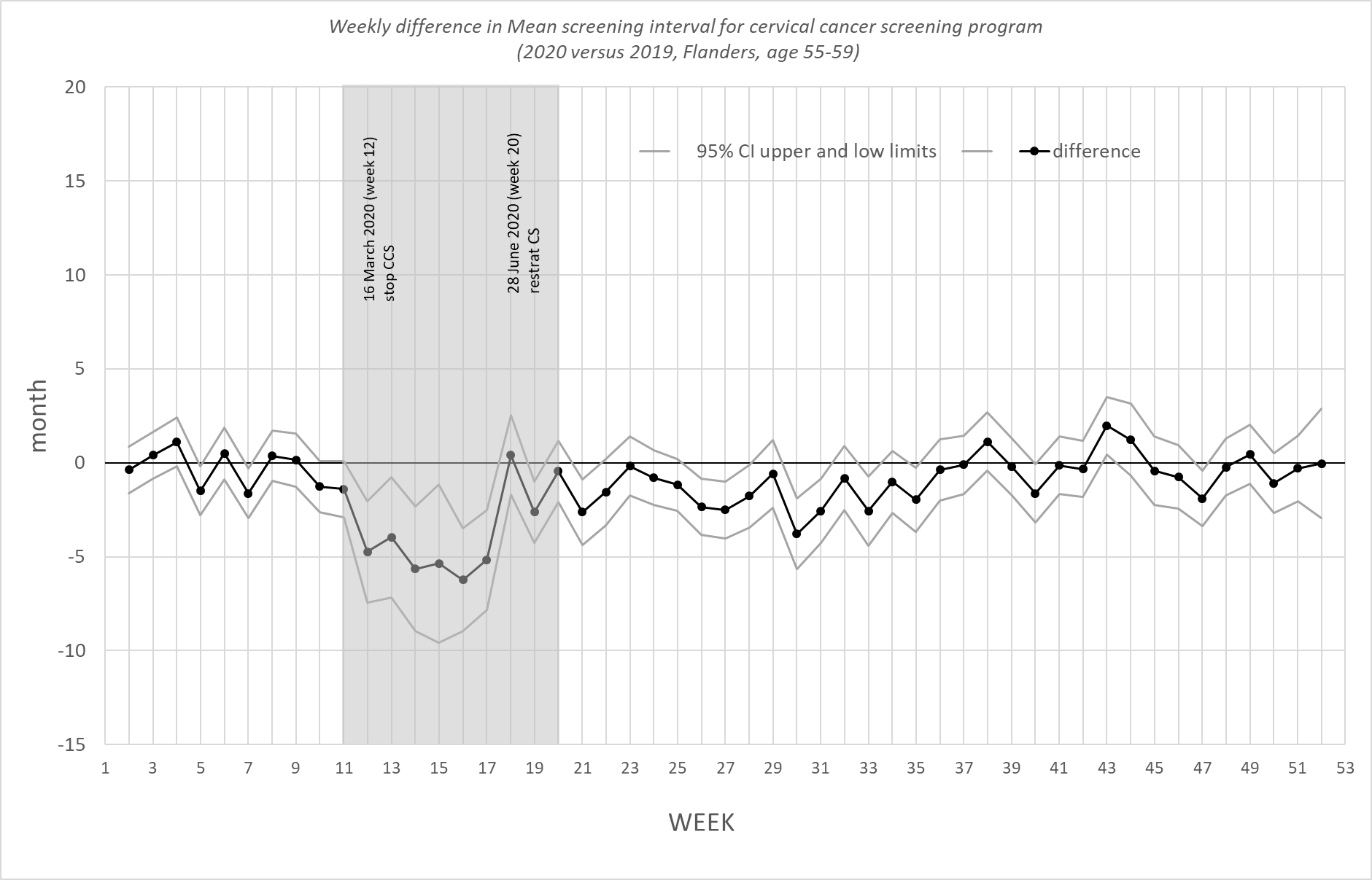 | 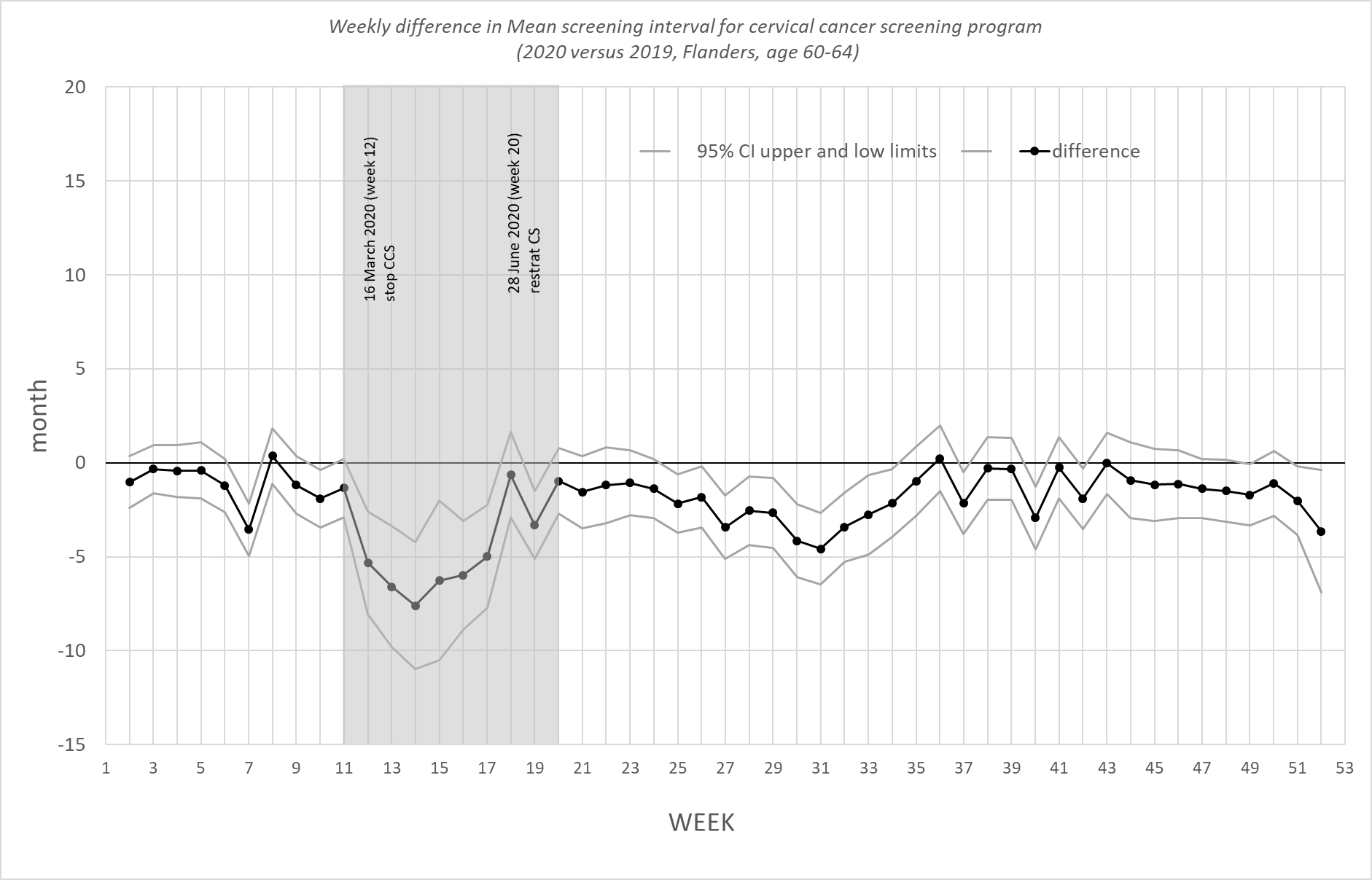 |
| g. 55-59 years old | h. 60-64 years old |
